# Analysis of pattern overlaps and exact computation of P-values of pattern occurrences numbers: case of Hidden Markov Models

**DOI:** 10.1186/s13015-014-0025-1

**Published:** 2014-12-16

**Authors:** Mireille Régnier, Evgenia Furletova, Victor Yakovlev, Mikhail Roytberg

**Affiliations:** INRIA, d’Estienne d’Orves 1, Palaiseau, 91120 France; Institute of Mathematical Problems of Biology, 142290, Institutskaya, 4, Pushchino, Russia; Pushchino State University, 142290, Prospect Nauki, 5, Pushchino, Russia; Laboratoire J.-V. Poncelet (UMI 2615), 119002, Bolshoy Vlasyevskiy Pereulok, 11, Moscow, Russia; National Research University “Higher School of Economics”, 101978, Myasnitskaya str., 20, Moscow, Russia; Moscow Institute of Physics and Technology, 141700, Institutsky pereulok, 11, Moscow Region, Dolgoprudny, Russia; CNRS, d’Estienne d’Orves 1, Palaiseau, 91120 France; LIX, Ecole Polytechnique, Batiment A. Turing, Palaiseau, 91128 France

**Keywords:** *P*-value, Pattern occurrences, PSSM (PWM), Hidden Markov model

## Abstract

**Background:**

Finding new functional fragments in biological sequences is a challenging problem. Methods addressing this problem commonly search for clusters of pattern occurrences that are statistically significant. A measure of statistical significance is the *P*-value of a number of pattern occurrences, i.e. the probability to find at least *S* occurrences of words from a pattern  in a random text of length *N* generated according to a given probability model. All words of the pattern are supposed to be of same length.

**Results:**

We present a novel algorithm SufPref that computes an exact *P*-value for Hidden Markov models (HMM). The algorithm is based on recursive equations on text sets related to pattern occurrences; the equations can be used for any probability model. The algorithm inductively traverses a specific data structure, an overlap graph. The nodes of the graph are associated with the overlaps of words from . The edges are associated to the prefix and suffix relations between overlaps. An originality of our data structure is that pattern  need not be explicitly represented in nodes or leaves. The algorithm relies on the Cartesian product of the overlap graph and the graph of HMM states; this approach is analogous to the automaton approach from JBCB 4: 553-569. The gain in size of SufPref data structure leads to significant improvements in space and time complexity compared to existent algorithms. The algorithm SufPref was implemented as a C++ program; the program can be used both as Web-server and a stand alone program for Linux and Windows. The program interface admits special formats to describe probability models of various types (HMM, Bernoulli, Markov); a pattern can be described with a list of words, a PSSM, a degenerate pattern or a word and a number of mismatches. It is available at http://server2.lpm.org.ru/bio/online/sf/. The program was applied to compare sensitivity and specificity of methods for TFBS prediction based on *P*-values computed for Bernoulli models, Markov models of orders one and two and HMMs. The experiments show that the methods have approximately the same qualities.

**Electronic supplementary material:**

The online version of this article (doi:10.1186/s13015-014-0025-1) contains supplementary material, which is available to authorized users.

## Background

The recognition of functionally significant fragments in biological sequences is a key issue in computational biology. Many functionally significant fragments are characterized by a set of specific words that is called a pattern and denoted  below. These patterns may represent different biological objects, such as transcription factor binding sites [1-3], polyadenylation signals [4], protein domains, etc. The functional fragments recognition problem can be solved by finding sequences in which the words from a given pattern are overrepresented. Defining a meaningful significance criteria for this overrepresentation is a delicate goal, that, in turn, requires a clarification of the probability model. A current criteria is the so-called *P*-value computed as the probability that a random sequence of length *N* contains at least *S* occurrences of a pattern. There are many methods for *P*-value computation designed for Bernoulli or Markov models. However, Hidden Markov models (HMM) were considered in only a few papers [5,6] despite the models being widely used in bioinformatics. This is a motivation to develop methods for *P*-value calculation with respect to HMMs.

Existing methods for *P*-value calculation can be divided into several groups and reviews of the methods can be found in [7-10]. Studies on word probabilities started as early as the eighties with the seed paper [11] that introduced basic word combinatorics and derived inductive equations for a single word and a uniform Bernoulli model. Some works in the same vein, reviewed in [12] followed for several words, multi-occurrences and extended probability models. The time complexity is proportional to the text length *N* and the desired number of occurrences *S*: computations are carried out by induction for *n* ranging over 1,…,*N* and, for a given *n*, by induction on the number of occurrences. Although these “mathematics-driven” approaches allow for mathematical formula derivation, the actual computation suffers from a combinatorial explosion when $|\mathcal {H}|$ or Markov order increase.

Later on, a first group of methods [13-17] formalized systematically these inductions by the introduction of bivariate generating functions. Coefficients are the *P*-values to be computed. Expectations and variances for the number of occurrences of the different words in pattern  can be expressed explicitly in terms of these generating functions [14,15,18]. Moreover, coefficients may be computed from the analytical expression, when it is available, or through a suitable manipulation of a functional equation, where the theoretical time complexity reduces to *S* log*N*. Nevertheless, computing the generating function, or the functional equation, requires the computation of a system of linear equations or, equivalently, the determinant of a matrix of polynomials of size $O(|\mathcal {H}|)$. It takes $O(|\mathcal {H}|^{3})$ operations and it is the main drawback of this approach.

A second group of methods computes asymptotics. They rely on convergence results to the normal law proved by [19] or [20]. An approximated *P*-value is derived, based on Gaussian approximations [21] or Poisson approximations [22-25]. Nevertheless, this approximation is not suitable for exceptional words, when the observed number of occurrences *S* significantly differs from the expected number. This was proved experimentally by [26] and theoretically [27]. Large deviation principles are used in [28,29] with a much better precision. Nevertheless, no computable formula are available for large sets.

A third group of methods revisits recursive *P*-value computation, with a *O*(*S*×*N*) time complexity. They avoid combinatorial explosion by a suitable use of appropriate data structures, tightly related to word overlap properties. Therefore, loss in time dependency to *N* or *S* is compensated by a gain on data structure size. A significant part of algorithms in this group are based on traversals of a specific graph. The graph may not be defined explicitly [30]. It can be based on the graph corresponding to the finite automaton recognizing the given pattern, see algorithms AHOPRO [31], SPATT [25,32] and REGEXPCOUNT [17]. MOTIFRANK [33] that is designed for first order Markov models makes use of suffix sets. In [25,32,34], a Markov chain embedding technique was suggested. Counting occurrences of regular patterns in random strings produced by Markov chains reduces to problems regarding the behavior of a first-order homogeneous Markov chain in the state space of a suitable deterministic finite automaton (DFA) [35,36]. In a recent paper [6], a probabilistic arithmetic automaton for computing *P*-values for a HMM was proposed. In this paper two algorithms were suggested. The first one has a time complexity *O*(|*Q*|^2^×*N*×*S*×|*Ω*|×|V|) and a space complexity *O*(|*Q*|×*S*×|*Ω*|), where |*Q*| is the number of states of the HMM, |*Ω*| is the number of states of the automaton recognizing the given pattern, |V| is the alphabet size. The second algorithm has a time complexity *O*(|*Q*|^3^×*log*(*N*)×*S*^2^×|*Ω*|^3^) and a space complexity *O*(|*Q*|^2^×*S*×|*Ω*|^2^). This algorithm uses the “divide and conquer” technique. The drawback is the lack of control on the number of states |*Ω*| when $|\mathcal {H}|$ increases. Finally, despite these great efforts, existing methods perform badly for rather big patterns. Besides this, most of the proposed algorithms are not implemented or implemented only for Bernoulli models or Markov models of small orders.

The present paper provides an algorithm supporting the HMM probability model. It assumes that all words have the same length *m* and that a HMM with |*Q*| states is given. It is a generalization of algorithm SUFPREF designed in [37] for Bernoulli models and Markov models of order *K*. It relies on recurrent equations based on an overlap graph, whose vertices are associated with the overlaps of words from , and edges correspond to the prefix and suffix relations between overlaps. The time complexity is $O(|Q|^{2} \!\times \! N \!\times \! S\! \times \! (|OV{(\mathcal {H})}|\! +\! |\mathcal {H}|))$ and the space complexity is $O(|Q|^{2} \!\times \! (|OV\!{(\mathcal {H})}|+|\mathcal {H}|)+|Q|\times S\times m \times |OV{(\mathcal {H})}|+m \times |\mathcal {H}|)$, where $|OV{(\mathcal {H})}|$ is the number of overlaps between the words from the pattern . In the case of a Markov model of order *K*, where *K* ≤ *m*, bounds for time and space above can be reduced to $O(N\! \times \! S\! \times \! (K\!\times \! |\mathrm {V}|^{K+1}+\! |OV{(\mathcal {H})}|\,+\,|\mathcal {H}|))$ and to $O(S\! \times \! K\! \times \! |\mathrm {V}|^{K+1}\,+\, S\! \times \! m \!\times \! |OV{(\mathcal {H})}|\,+\,m\!\times \!|\mathcal {H}|)$, respectively. Algorithm SUFPREF is implemented as a Web-server, see http://server2.lpm.org.ru/bio/online/sf/, and a stand-alone program for Windows and Linux. The program is available by request from the authors.

The paper is organized as follows. Basic notions on word overlaps are introduced, that lead to an overlap graph that is the main data structure to be used. Then, one recalls the Hidden Markov models definition. Main text sets are defined and equations for their probabilities are derived. The next section describes the algorithm SUFPREF that computes these equations using the overlap graph as a main data structure. Then, the space and time complexities are analyzed and our algorithm is compared with other methods [3,24,31,38,39]. Finally, usage of *P*-values for TFBS prediction is discussed.

## Overlap words

Our approach strongly relies on overlaps of words from a given pattern. In this section we provide necessary definitions for these overlaps, following the notations of [37]. The main deference is in definition of overlap graph, see definition 3. By definition from [37], overlap graph has additional nodes (leaves) that correspond to the words from the pattern . In the present paper overlap graph has deep edges instead of the nodes. This modification is not affect on upper bounds of time and space complexity. But in practice it gives significant improvements.

### **Definition1**.

Given a pattern  over an alphabet V, a word *w* is an *overlap* (an overlap word) for  if there exist words H and F in  such that *w* is a proper suffix of H and *w* is a proper prefix of F. The set of overlaps of the pattern  is denoted $OV(\mathcal {H})$.

### **Example1**.

Let  be the set

$$\begin{array}{@{}rcl@{}} \mathcal{H}\! &=& \!\{\text{ACAGCTA, ACATATA, CTTTCGC, TACCACA}\}. \end{array} $$

The overlap set is

$$ OV(\mathcal{H}) = \{\epsilon,\text{A, ACA, C, TA}\}. $$

### **Notation**.

Below we will use the following notations: 1) *w*, for an overlap from $OV{(\mathcal {H})}$; 2) H, for a word from the pattern ; 3) *v*, for a word from $OV{(\mathcal {H})} \cup \mathrm {H}$.

### **Notation**.

For an overlap *w* in $OV{(\mathcal {H})}$, one denotes

$$\mathcal{H}(w) = \{\mathrm{H} \in \mathcal{H} \ | \ \mathrm{H}\ \text{ends with}\; w \} ~~, $$

with the convention $\mathcal {H} (\epsilon) = \mathcal {H}$.

### **Notation**.

$ x^{\prime } \sqsubseteq x$ (*x*^′^⊂*x*) means that *x*^′^ is a suffix (proper suffix) of *x*; *x*^′^≼*x* (*x*^′^≺*x*) means that *x*^′^ is a prefix (proper prefix) of *x*. The elements of $OV{(\mathcal {H})}$ that are proper prefixes (respectively suffixes) of a given word are totally ordered. The empty string is the minimal element. The maximal elements are crucial for our algorithms and data structures.

### **Definition2**.

Given a word *v* in $OV{(\mathcal {H})} \cup \mathcal {H} \setminus \{\epsilon \}$, one denotes

$$\begin{array}{@{}rcl@{}} lpred(v) &=& \max \{x| x \in OV{(\mathcal{H})} \;\text{and}\; x \prec v \} ; \\ rpred(v) &=& \max \{x| x \in OV{(\mathcal{H})} \;\text{and}\; x \subset v \}. \end{array} $$

Two words H and F from the pattern  are called *equivalent* if they satisfy

$$\begin{array}{@{}rcl@{}} lpred (\mathrm{H}) &=& lpred (\mathrm{F}), \\ rpred (\mathrm{H}) &=& rpred (\mathrm{F}). \end{array} $$

### **Notation**.

Given two words *x* and *w* in $OV{(\mathcal {H})}$, let H^∗^(*x*,*w*) denote the equivalence class consisting of all words $\mathrm {H} \in \mathcal {H}$ such that *lpred*(H)=*x* and *rpred*(H)=*w*.

One notes, for a word H in H^∗^(*x*,*w*),

(1) $$ \begin{aligned} lpred (\mathrm{H}^{\ast}(x,w)) &= lpred (\mathrm{H}) \;\text{and}\; rpred (\mathrm{H}^{\ast}(x,w))= \\&= rpred (\mathrm{H}). \end{aligned}  $$

Let $\mathcal {P}(\mathcal {H})$ denote the set of all equivalence classes on .

### **Example2**.

Consider the pattern  from the previous example.

1. For the overlap $\text {ACA} \in OV(\mathcal {H})$, *lpred*(ACA)=A, because A is the maximal prefix of ACA that is overlap. Analogously, *rpred*(ACA)=A.
2. The words ACAGCTA and ACATATA from the pattern are equivalent because
  $$lpred (\text{ACAGCTA}) = lpred(\text{ACATATA}) = \text{ACA and } $$
  $$rpred (\text{ACAGCTA}) = rpred(\text{ACATATA}) = \text{TA}. $$
  These words are in the class $\mathcal {H}^{*}(\text {ACA, TA})$. The partition $\mathcal {P}(\mathcal {H})$ consists of three classes: $\mathcal {H}^{*}(\text {ACA, TA}) = \{\text {ACAGCTA, ACATATA}\}$, $\mathcal {H}^{*}(\text {C, C}) = \{\text {CTTTCGC}\}$ and $\mathcal {H}^{*}(\text {TA, ACA}) = \{\text {TACCACA}\}$.

Order relations are commonly associated to *oriented graphs*.

### **Definition3**.

The overlap graph of a given pattern  is an oriented graph where the set of nodes is $OV{(\mathcal {H})}$ and the set of edges, $E(\mathcal {H})$, contains the left, right and deep edges, that are defined as follows:

- A left edge links node *x* to node *w* iff *x*=*lpred*(*w*);
- A right edge links node *x* to node *w* iff *x*=*rpred*(*w*);
- A deep edge links node *x* to node *w* iff there exists a non-empty class H^∗^(*x*,*w*) in $\mathcal {P}(\mathcal {H})$.

It is denoted *OvGraph*.

The root is the node corresponding to the empty word.

### **Definition4**.

An overlap $w \in OV{(\mathcal {H})}$ is called a left deep node, respectively a right deep node, if there exists a word $\mathrm {H} \in \mathcal {H}$ such that *w*=*lpred*(H), respectively *w*=*rpred*(H). The sets of all left and right deep nodes are denoted by $DLOV(\mathcal {H})$ and $DROV(\mathcal {H})$.

### **Notation**.

For a right deep node $r \in DROV(\mathcal {H})$, one denotes

$$\widetilde{\mathcal{H}}(r) = \{\mathrm{H} \in \mathcal{H} \ | \ r =rpred(\mathrm{H}) \}. $$

Below we will use *r* for notation of a right deep node.

### **Definition5**.

Let *v* be in $(OV{(\mathcal {H})} \cup \mathcal {H}) \setminus \epsilon $.

The set of non-empty prefixes of *v* (including *v*) that belong to $OV{(\mathcal {H})}$ is denoted by *OverlapPrefix*(*v*). For any prefix *x* in *OverlapPrefix*(*v*), let *Back*(*x*,*v*) denote the suffix of *v* that satisfies the equation

$$v = x \cdot Back(x,v). $$

Let *Back*(*v*) denote *Back*(*lpred*(*v*),*v*).

Also for $\mathrm {H}^{\ast }(w,r) \in \mathcal {P}(\mathcal {H})$ we denote

$$Back(\mathrm{H}^{\ast}(w,r)) = \bigcup\limits_{\mathrm{H} \in \mathrm{H}^{\ast}(w,r)}Back(\mathrm{H}). $$

### **Remark**.

One can ascribe to each deep edge (*w*,*r*) the class H^∗^(*w*,*r*) and to each left edge (*lpred*(*w*),*w*) a word label *Back*(*w*).

### **Example3**.

**Figure 1:**
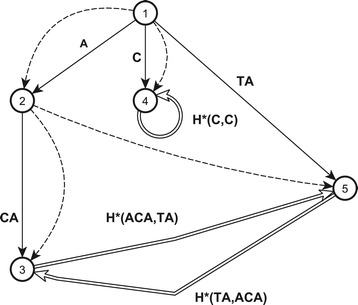
**Overlap graph for pattern** $\boldsymbol{\mathcal {H}} = \{\text {ACAGCTA, ACATATA}$ **, CTTTCGC, TACCACA}.** Nodes are the elements of $OV{(\mathcal {H})}$. The node with the index number “1” corresponds to *ε*, it is the root. The left edges are shown by continuous straight lines, right edges are shown by dashed lines and deep edges are shown by double lines. Each left edge (*l* *p* *r* *e* *d*(*w*),*w*), where $w \in OV{(\mathcal {H})}$, is labeled with *B* *a* *c* *k*(*w*). For example, edge (2,3) corresponding to the pair of overlaps (A, ACA) is labeled with *B* *a* *c* *k*(ACA)=CA. A deep edge (*w*,*r*) corresponds to equivalence class $\mathcal {H}^{*}(w,r)$. The right edges (*w*,*r* *p* *r* *e* *d*(*w*)) are not labeled.

The overlap graph for the pattern $\mathcal {H} = \{\text {ACAGCTA, ACATATA, CTTTCGC, TACCACA}\}$ is shown in Figure 1. The nodes of the graph correspond to the overlaps from the set $OV(\mathcal {H}) = \{\epsilon, \text {A, ACA, C, TA}\}$. The index numbers of nodes are the index numbers of overlaps in the prefix order. The graph has four left edges (shown by straight lines), five right edges (shown by dashed lines) and three deep edges (shown by double lines).

## Text sets

The computation of *P*-values will be done by induction on the text length *n* (*n*=1,…,*N*), and, for each given *n*, by induction on the number of occurrences *s* (*s*=1,…,*S*).

It relies on formulas introduced in [37], that in turn was based on the ideas from [12,13]. In [37] we give formulas for *P*-values computation for Bernoulli and Markov models. In the present paper we introduce equations on texts sets that underlie these formulas. Using these equations one can derive formulas for *P*-value computation for different probabilities models. Also these equations take into account improvements in the overlap graph structure, see section “Overlap words”.

### **Definition6**.

Let  be a pattern.

$$ \begin{aligned} B(n,s) =& \{t \in \mathrm{V}^{n} | t \;\text{contains at least}\; s\\ &\text{occurrences of the pattern}\; \mathcal{H}\}. \end{aligned}  $$

By convention, *B*(*n*,0)=*V*^*n*^.

### **Definition7**.

Given a right deep node $r \in DROV(\mathcal {H})$, one defines, for *s*=1,…,*S*,*S*+1

(2) $${} \begin{aligned} E(n,s,r) =&\{t \in {V}^{n} | t \;\text{contains at least}\; s \;\text{occurrences of}\; \mathcal{H}\,\text{\&}\\ &\text{\&}\;t \;\text{ends with}\; \mathrm{H} \in\widetilde{\mathcal{H}}(r)\} \end{aligned}  $$

These sets are called *E-sets*.

### **Definition8**.

Let ${w}\! \in \! OV{(\mathcal {H})}$, one defines, for *s* = 1,…,*S*

(3) $${} \begin{aligned} R(n,s,w) =&\{t \in {V}^{n} | t \;\text{contains exactly}\; s \;\text{occurrences of}\; \mathcal{H}\,\text{\&}\\ &\;\text{\&}\; t \;\text{ends with}\; \mathrm{H} \in \mathcal{H}(w)\} \end{aligned}  $$

These sets are called *R-sets*.

### **Remark**.

We remark that

$${} \begin{aligned} R(n,s,\varepsilon) =& \{t \in \mathrm{V}^{n}| t \;\text{contains exactly}\; s \;\text{occurrences of}\; \mathcal{H}\,\text{\&}\\ & \text{\&}\; t \;\text{ends with}\; \mathrm{H} \in \mathcal{H} \}  \end{aligned}  $$

Note, if *t*∈*E*(*n*,*s*,*r*) then *t* ends with a word H from $\mathcal {H}(r)$, where *r*=*rpred*(H). In contrast, if *t*∈*R*(*n*,*s*,*w*) then *t* ends with a word H from $\mathcal {H}(w)$, i.e. *w* is a suffix of H.

### **Example4**.

Consider the pattern $\mathcal {H} = \{\mathrm {ACAGCTA, ACATATA, CTTTCGC, TACCACA}\}$ from the example 1. And consider the text *t*_1_ = **CTTTCGC**CGAATC**ACAGCTA**. The texts is of length 20, contains exactly 2 occurrences of  (the occurrences are given in bold) and ends with ACAGCTA. Obviously, *rpred*(ACAGCTA)=TA. Thus *t*_1_ is in *B*(20,1), *B*(20,2), *E*(20,1,TA), *E*(20,2,TA), *R*(20,2,TA), *R*(20,2,A) and *R*(20,2,*ε*).

### **Example5**.

Consider the pattern  from the previous examples and the set *E*(20,2,TA). A text *t* from *E*(20,2,TA) is of length 20, has at least 2 occurrences of  and ends with a word H from  such that *rpred*(H) = TA. Obviously, H is ACAGCTA or ACATATA. The words ACAGCTA and ACATATA are from the same class H^∗^(ACA,TA). For example, texts *t*_1_=**CTTTCGC**CGAATC**ACAGCTA**, *t*_2_=**CTTTCGC**GG*TACC****ACA*TATA**, *t*_3_=**TACC*ACA****TATA*CC**ACAGCTA**, *t*_4_=ACGTTTCCA*TACC****ACA*GCTA**, *t*_5_ = ACTAAG*ACAGCT****A*CATATA** are in *E*(20,2,TA). The occurrences of  are given in bold or italic.

### **Definition9**.

Given a right deep node $r \in DROV(\mathcal {H})$, one defines, for *s*=1,…,*S*

(4) $$ RE(n,s,r)= \{t \in R(n,s,r)| t \;\text{ends with}\; \mathrm{H} \in \widetilde{\mathcal{H}}(r) \}.\;\;\;  $$

Remark that

(5) $$  RE(n,s,r)=E(n,s,r)\setminus E(n,s+1,r).  $$

### **Example6**.

Consider the pattern

$$\begin{aligned} \mathcal{H}_{1} =& \mathcal{H} \cup \text{ATAGTCG} = \{\text{ACAGCTA, ACATATA,} \\&\text{ATAGTCG, CTTTCGC, TACCACA}\}, \end{aligned} $$

where  is the pattern from the previous examples. Obviously, $ OV(\mathcal {H}_{1}) = \{\epsilon,\text {A, ACA, ATA, C, TA}\}$. Consider the texts *t*_1_=**CTTTCGC**CGAATC**ACAGCTA** and *t*_5_=ACTAAG*ACAGCT****A*CATATA**. The texts *t*_1_ and *t*_5_ belong to *R*(20,2,TA) because the texts: 1) have length 20; 2) contain exactly two occurrences of $\mathcal {H}_{1}$ and 3) end with the words from $\mathcal {H}_{1}(\text {TA})$, here TA is the suffix of the words. Also the text *t*_1_ is in *RE*(20,2,TA) because it ends with ACAGCTA, and *rpred*(ACAGCTA)=TA. In contrast, *t*_5_ is not in *RE*(20,2,TA) because it ends with ACATATA, and *rpred*(ACATATA)=ATA.

The following proposition gives the inductive relations allowing effective computation of probabilities of *R*-sets.

### **Proposition1**.

Let $w \in OV{(\mathcal {H})}$. If *w* is a deep right node, i.e. *w*=*rpred*(H) for a word $\mathrm {H}\in \mathcal {H}$, then

(6) $$  R(n, s, w) = RE(n, s, w) \cup \left(\bigcup\limits_{x \in OV{(\mathcal{H})}: w=rpred(x)}R(n, s, x)\right)\!,  $$

otherwise,

(7) $$ R(n, s, w) = \bigcup_{x \in OV{(\mathcal{H})}: w=rpred(x)}R(n, s, x).  $$

The proof follows from the definition of *R*-sets.

### **Example7**.

Lets illustrate the proposition 1 with the data from the example 6. As we have seen before, *t*_1_,*t*_5_∈*R*(20,2,TA). Further, *t*_1_∈*RE*(20,2,TA), and *t*_5_∈*R*(20,2,ATA). Here, TA=*rpred*(ATA). Also note, *R*(20,2,ATA)=*RE*(20,2,ATA).

### **Remark**.

For given *n* and *s*, we have to compute the probabilities of sets *R*(*n*,*s*,*w*) for all $w \in OV{(\mathcal {H})}$. The equations (6) and (7) allow us to do this by recursive traversal of $OV{(\mathcal {H})}$ from the leaves (deep nodes) of *OvGraph* to the root according to the right edges. The calculation starts from probabilities of *RE*-sets found according to the equation (5).

Below we introduce *D*-sets and give the equations for *D*-sets, *R*-sets and *E*-sets leading to recursive equations for *E*-sets probabilities. The *D*-sets defined below consist of texts of length *n* containing at least *s* occurrences of the pattern , ending with a given non-empty overlap word *w* that has a common part with the *s*-th occurrence of the pattern .

### **Definition10**.

Let $w \in OV{(\mathcal {H})}$, *w*≠*ε*, *k*≥1.

(8) $$\begin{array}{@{}rcl@{}} D(k, s, w) &=& \{t \in B(k,s) |w \;\text{is a suffix of}\; t \; \text{\&}  \\ &&\text{\&} \,s \;\text{-th occurrence of the pattern}\; \mathcal{H} \;\text{intersects} \\&&\text{the suffix}\; w \}. \end{array} $$

By definition, *D*(*k*,*s*,*ε*)=*∅*.

### **Notation**.

Below we will use the following notations: 1) *len*(*x*), for the length of a word *x*; 2) |*M*|, for the number of words in a set of words *M*.

### **Notation**.

For a prefix $w \in OV{(\mathcal {H})}$ and any integer *n*, one denotes

$$  k(n,w) = n-m+len(w),   $$

where *m* is the length of words from .

### **Example8**.

Let *n*=20 and *s*=2. Consider the pattern $\mathcal {H} = \{\text {ACAGCTA, ACATATA, CTTTCGC, TACCACA}\}$ and the texts *t*_4_=ACGTTTCCA*TACC****ACA*GCTA**, *t*_5_=ACTAAG*ACAGCT****A*CATATA** from the example 5. In the both cases, the first occurrence of  intersects the ending occurrence of . The texts end with words from the class H^∗^(ACA,TA)={ACAGCTA, ACATATA}.

Consider the overlap *w*=ACA. Then *k*(*n*,*w*)=16. Consider the prefixes *t*_4_[ 1,16]=ACGTTTCCA*TACC****ACA*** and *t*_5_[ 1,16]=ACTAAG*ACAGCT****A*CA** of the texts. For these prefixes we have: (1) their length is 16; (2) the prefixes end with ACA; (3) the prefixes have at least *s*−1=1 occurrence of  and (4) the first occurrence of  intersects the suffix ACA. Thus the prefixes *t*_4_[ 1,16] and *t*_5_[ 1,16] are in *D*(16,1,ACA). Further, *t*_4_ and *t*_5_ are in *D*(16,1,ACA)·*Back*(H^∗^(ACA,TA)), where *Back*(H^∗^(ACA,TA))={GCTA,TATA}. Note, that *t*_5_[ 1,14] also belongs to *D*(14,1,A).

The next propositions describe the relation between *D*-sets and *R*-sets.

### **Proposition2**.

Let $w \in OV{(\mathcal {H})}$, *w*≠*ε*. Then

(9) $$  \begin{aligned} &D(k(n,w), s, w)=\\ &\!\!= \!\!\bigcup\limits_{x \in Overlap\,\text{Pr}\,efix(w)}\!\!\!\!\!\!\!\!\!\!\!\!\! \!R(k(n,\!w)\!- \!len(Back(x,\!w)\!),\!s,\!x) \!\cdot\! Back(x,\!w). \end{aligned}  $$

**Proof:** [see Additional file 1].

Informally speaking, *x* is the common part of the *s*-th occurrence of the pattern  in the text *t*∈*D*(*k*(*n*,*w*),*s*,*w*) and the suffix *w* of the text *t*. Remark that according to the definition 5: (1) *ε* is not in *OverlapPrefix*(*w*), (2) *w* is in *OverlapPrefix*(*w*).

### **Proposition3**.

Let $w \in OV{(\mathcal {H})}\setminus \epsilon $, *n*≥*m*,*s*≥1. Then

(10) $$ \begin{aligned} D(k(n,w), s, w) =&\,D(k(n,lpred(w)), s, lpred(w))\times \\&\times Back(w) \cup R(k(n,w),s, w) ~~. \end{aligned}  $$

**Proof:** Follows from the proposition 2 [see Additional file 1].

### **Corollary1**.

If *lpred*(*w*)=*ε* then *D*(*n*,*s*,*w*)=*R*(*n*,*s*,*w*).

One observes that, whenever *n*<*m*, *B*(*n*,*s*)=*∅*, and for all $w \!\in \! OV{(\mathcal {H})}$ and $r \!\in \! DROV(\mathcal {H})$, *R*(*n*,*s*,*w*) = *E*(*n*,*s*,*r*) =*∅*.

Now we are ready to formulate the main theorem of the section. The theorem gives recursive equations for *B*-sets and *E*-sets. The main equations (13)–(15) are based on the following observation. The set *E*(*n*,*s*+1,*r*), *s*≥1, can be divided in two disjoint sets: *F*(*n*,*s*+1,*r*) and *C*(*n*,*s*+1,*r*). The set *F*(*n*,*s*+1,*r*) consists of such words that *s*-th occurrence of the pattern  does not intersect the ending occurrence of . And *C*(*n*,*s*+1,*r*) consists of those texts *t* from *E*(*n*,*s*+1,*r*) that *s*-th occurrence of  in *t* intersects the ending occurrence of .

### **Theorem1**.

Let *n*≥*m*, *s*≥1 and $r \in DROV(\mathcal {H})$, i.e. *r* is a right deep node.

1. Sets *B*(*n*,*s*) and *E*(*n*,*s*,*r*) meet the following equations:
  (11) $$\begin{array}{@{}rcl@{}} B(n,s) &=& B(n-1,s) \cdot \mathrm{V} \cup R(n,s,\epsilon)  \enspace \end{array} $$
  (12) $$\begin{array}{@{}rcl@{}} E(n,1,r) &=& \mathrm{V}^{n-m}\cdot \widetilde{\mathcal{H}}(r) \enspace  \end{array} $$
  (13) $$\begin{array}{@{}rcl@{}} F(n,s+1,r) &=& B(n-m,s) \cdot \widetilde{\mathcal{H}}(r)\enspace  \end{array} $$
  (14) $$\begin{array}{@{}rcl@{}} C(n,s+1,r) &=& \bigcup_{w: (w,r) \text{ is a deep edge}} D(k(n,w), s,w)\times ~~~\\ &&\times Back(\mathrm{H}^{\ast}(w,r)) \enspace  \end{array} $$
  (15) $$\begin{array}{@{}rcl@{}} E(n,s+1,r) &=& F(n,s+1,r) \cup C(n,s+1,r) \enspace  \end{array} $$
  Note, that (*w*,*r*) is a deep edge iff $\mathrm {H}^{*}(w,r) \in \mathcal {P}(\mathcal {H}) $, see definition 3.
2. Unions (11), (14) and (15) are disjoint, i.e.
  $$B(n-1,s) \cdot \mathrm{V} \cap R(n,s,\epsilon) = \emptyset ~~; $$
  if (*w*,*r*)≠(*v*,*x*) then
  $$\begin{aligned} &D(k(n,w), s,w) \cdot Back(\mathrm{H}^{\ast}(w,r)) \cap D(k(n,v), s,v)\times~ \\&\quad\times Back(\mathrm{H}^{\ast}(v,x))= \emptyset; \end{aligned} $$
  $$F(n,s+1,r) \cap C(n,s+1,r) = \emptyset. $$

### **Example9**.

The statements (13)–(15) can be illustrated with the data from the examples 5 and 8. Let *n*=20, *s*=1, *r*=TA. Then (15) can be rewritten as

$$E(20, 2, \text{TA}) = F(20, 2, \text{TA}) + C(20, 2, \text{TA}). $$

Consider the texts *t*_1_,…,*t*_5_ from the example 5.

In each of the texts *t*_1_,*t*_2_,*t*_3_ the ending occurrence of the pattern does not intersect the first occurrence. Therefore the texts are in *F*(20,2,TA). Note, that the ending occurrence ACATATA of the pattern in *t*_2_ intersects the second occurrence but not the first. Consider the prefixes of *t*_1_, *t*_2_ and *t*_3_ of length *n*−*m*=20−7=13, *t*_1_[ 1,13]=**CTTTCGC**CGAATC, *t*_2_[ 1,13]=**CTTTCGC**GGTACC and *t*_3_[ 1,13]=**TACC*ACA****TATA*CC. The prefixes contain at least one occurrence of , i.e. the prefixes are in *B*(13,1). Thus $t_{1},t_{2}, t_{3} \in B(13,1) \cdot \widetilde {\mathcal {H}}(\text {TA})$, that is in agreement with the statement (13) of the theorem. Obviously,

$$\begin{aligned} \widetilde{\mathcal{H}}(\text{TA}) &= \mathcal{H}(\text{TA}) = \mathcal{H}^{*}(\text{ACA, TA})= \\&= \{\text{ACAGCTA, ACATATA}\}. \end{aligned} $$

In contrast, in each of the texts *t*_4_ and *t*_5_ the last occurrence of the pattern intersects the first occurrence. Therefore the texts *t*_4_,*t*_5_∈*C*(20,2,TA). According to the example 7, the texts *t*_4_,*t*_5_ are in *D*(16,1,ACA)·*Back*(H^∗^(ACA,TA)), that illustrates the statement (14) of the theorem.

Note, there is only one overlap *w* such that $\mathcal {H}^{*}(w, \text {TA})\! \neq \emptyset $, that is *w*=ACA. Thus

$$C(20, 2, \text{TA}) = D(16,1,\text{ACA}) \cdot Back(\mathrm{H}^{*}(\text{ACA,TA})). $$

**Proof:**

1. Consider statement (11). A text *t* is in *B*(*n*,*s*) if and only if either its prefix of length *n*−1 contains at least *s* occurrences of  or a *s*-th occurrence H from  ends at position *n*. In the first case, *t* is in *B*(*n*−*m*,*s*)·V. In the second case, text *t* belongs to *R*(*n*,*s*,*ε*). The two cases are mutually exclusive; therefore *B*(*n*,*s*) is a disjoint union and (11) is proved.
2. The statement (12) directly follows from the definition of *E*(*n*,1,*r*).
3. Consider the statement (13).
  - First, we prove that $F(n,s+1,r) \subseteq B(n-m,s) \cdot \widetilde {\mathcal {H}}(r)$. When a text *t* is in *F*(*n*,*s*+1,*r*), it ends with a word $\mathrm {H} \in \mathcal {H}$ such that *r*=*rpred*(H), i.e. $\mathrm {H} \in \widetilde {\mathcal {H}}(r)$. The last occurrence H of the pattern does not intersect the *s*-th occurrence in the text *t*. Thus the prefix of *t* of length *n*−*m* contains at least *s* occurrences of , i.e. it is in *B*(*n*−*m*,*s*), where *m* is the length of pattern words. Therefore *t* is in $B(n-m,s)\cdot \widetilde {\mathcal {H}}(r)$.
  - Obviously, if $t \in B(n-m,s)\cdot \widetilde {\mathcal {H}}(r)$ then
    *t* has the length *n*;
    *t* contains at least *s*+1 occurrences of the pattern ;
    *s*-th occurrence of  lies on the prefix of *t* of length *n*−*m*, i. e. it does not intersect the last occurrence;
    *t* ends with $\mathrm {H} \in \widetilde {\mathcal {H}}(r)$.
    Therefore *t*∈*F*(*n*,*s*+1,*r*).
4. Consider the statement (14). Let *Y* denote the right side of equation (14).
  - Prove that *C*(*n*,*s*+1,*r*)⊆*Y*. If a text *t* is in *C*(*n*,*s*+1,*r*) then it ends with a word $\mathrm {H} \in \widetilde {\mathcal {H}}(r)$. The last occurrence H intersects the *s*-th occurrence of the pattern in the text *t*. Let H_1_ be the *s*-th occurrence of  in *t*, and *x* be the overlap between H_1_ and H in *t*. Obviously, *x*∈*OverlapPrefix*(*w*), where *w*=*lpred*(H), see definition 5 of *OverlapPrefix*(*w*). The prefix of *t* of length *k*(*n*,*x*), where *k*(*n*,*x*)=*n*−*m*+*len*(*x*), contains exactly *s* occurrences of  and ends with H_1_, where $\mathrm {H}_{1} \in \mathcal {H}(x)$. By definition of *R*-sets, the prefix is in *R*(*k*(*n*,*x*),*s*,*x*). Therefore *t*∈*R*(*k*(*n*,*x*),*s*,*x*)·*Back*(*x*,H). Observing that
    $$Back(x,\mathrm{H}) =Back(x, w) \cdot Back(\mathrm{H}) $$
    we obtain
    $$t \in R(k(n,x), s, x) \cdot Back(x, w)\cdot Back(\mathrm{H}). $$
    Note, *k*(*n*,*x*)=*k*(*n*,*w*)−*len*(*Back*(*x*,*w*)), where *len*(*Back*(*x*,*w*))=*len*(*w*)−*len*(*x*).
    According to the proposition 2,
    $$\begin{aligned} &R(k(n,w) - len(Back(x,w)), s, x) \cdot Back(x, w) \subseteq \\ &\quad\subseteq D(k(n,w),s,w). \end{aligned} $$
    Thus
    $$t \in D(k(n,w),s,w) \cdot Back(\mathrm{H}). $$
    Note, if H∈H^∗^(*w*,*r*) then *Back*(H)⊆*Back*(H^∗^(*w*,*r*)). Therefore,
    $$\begin{aligned} &D(k(n,w),s,w) \cdot Back(\mathrm{H}) \subseteq D(k(n,w),s,w)\times \\&\quad\times Back(\mathrm{H}^{\ast}(w,r)). \end{aligned} $$
    This yields that *t*∈*Y*.
  - Proof that *Y*⊆*C*(*n*,*s*+1,*r*). Let *t*∈*Y*, i.e *t*∈*D*(*k*(*n*,*w*),*s*,*w*)·*Back*(H^∗^(*w*,*r*)). By the definition of *D*-sets,
    *t* has the length *n*;
    *t* contains at least *s*+1 occurrences of the pattern;
    *s*-th occurrence intersects (*s*+1)-th occurrence of ;
    *t* ends with $\mathrm {H} \in \widetilde {\mathcal {H}}(r)$.
    Thus *t*∈*C*(*n*,*s*+1,*r*).
5. The statement (15) follows from the definitions of *F* and *C*-sets.

### **Notation**.

Given two integers *n* and *s*, and a class H^∗^(*w*,*r*), one introduces

(16) $$  C^{\prime}(n,s+1, w,r) = D(k(n,x), s,w) \cdot Back(\mathrm{H}^{\ast}(w,r)) ;\,\,\,\,\,  $$

Obviously,

(17) $$  C(n,s+1,r) = \bigcup\limits_{w:~~(w,r) \;\text{is a deep edge}} C^{\prime}(n,s+1, w,r).\;\;\;\;  $$

### **Remark**.

The unions in equations (11), (14), (15) and (17) are disjoint. Therefore the probability of the set in the left part of an equation is the sum of probabilities of sets in the right side.

## Probability models

We suppose that the probability distribution is described by a Hidden Markov Model (HMM). In this section, we recall some basic notions about HMMs and introduce the needed notations. In fact, it is shown in [6] that our definition is equivalent to the classical definition of HMM [40].

### **Definition11**.

A HMM *G* is a triple *G*=〈*Q*,*q*_0_,*π*〉, where *Q* is the set of states, *q*_0_∈*Q* is an initial state, and *π* is a function: *Q*×V×*Q*→ [ 0,1] such that *π*(*q~*,*a*,*q*) is the probability, being in state *q~*, to generate symbol *a* and traverse to state *q*. For any state *q~* in *Q*, the function *π* meets the condition:

(18) $$ \sum\limits_{a \in \mathrm{V}} \sum\limits_{q \in Q} \pi (\textit{\~{q}},a,q) =1 ~~.  $$

A HMM *G* is called *deterministic* if for any (*q~*,*a*) in *Q*×V there is only one state *q* such that *π*(*q~*,*a*,*q*)>0. In this case the function *π* can be described with two functions:

1. a transition function *ϕ*:*Q*×V→*Q*;
2. a probability function *ρ*:*Q*×V→ [ 0,1].

Namely, *ϕ*(*q~*,*a*) is equal to the unique state *q* such that *π*(*q~*,*a*,*q*)>0 and *ρ*(*q~*,*a*) is *π*(*q~*,*a*,*q*).

A HMM *G*=〈*Q*,*q*_0_,*π*〉 can be represented as a graph where *Q* is the set of vertices. Each edge is assigned with a label *a*∈V and with a probability *p*∈(0;1]. There exists an edge from *q~* to *q* with the label *a* and probability *p* iff *π*(*q~*,*a*,*q*)>0 and *p*=*π*(*q~*,*a*,*q*). The graph is called the traversal graph of HMM *G*.

### **Definition12**.

Let *h* be a path in the traversal graph of the HMM *G*. The label of *h* is the concatenation of the labels of edges that constitute the path *h*. The probability *Prob*(*h*) of a path *h* is the product of the probabilities of the edges that constitute the path *h*.

### **Definition13**.

The probability *Prob*(*t*) of a word *t* with respect to the HMM *G* is the sum of probabilities of all paths that start in the initial state *q*_0_ and have the label *t*.

Let *q* and *q~* belong to *Q* and *t* be a word. By definition, the probability *Prob*(*q~*,*t*,*q*) to move from the state *q~* to the state *q* with the emitted word *t* is the sum of probabilities of all paths starting in the state *q~*, ending in the state *q* and having the word label *t*.

To describe effective algorithms related to HMMs, we need the notion of reachability.

### **Definition14**.

Given a state *q~* and a word *t*, we define

$$ReachState (t,\textit{\~{q}}) =\{q | Prob(\textit{\~{q}}, t, q) > 0 \}. $$

Given a state *q* and a string *t*, we define

$$StartState (t,q) =\{\textit{\~{q}} | Prob(\textit{\~{q}}, t, q) > 0 \}. $$

A state *q* is called *t*-reachable from a state *q~* if and only if *Prob*(*q~*,*t*,*q*)>0.

### **Definition15**.

For a given word *t*, *AllState*(*t*)is the set of states that are reachable from initial state *q*_0_ by at least one text with suffix *t*. For a set of words *M*,

$$AllState(M) = \bigcup\limits_{t\in M} AllState(t). $$

### **Remark**.

(19) $$ AllState(t) = \bigcup\limits_{t' \in \mathrm{V}^{*}.t} ReachState(t',q_{0}).  $$

### **Definition16**.

Let *w* be an overlap word. We denote by *PriorState*(*w*,*q*) the set of states *q~*∈*AllState*(*lpred*(*w*))such that *q* is *Back*(*w*)-reachable from *q~*, i.e.

$$\begin{aligned} PriorState(w,q) =&\, AllState(lpred(w))\cap\\&\cap StartState(Back(w),q); \end{aligned} $$

Analogously, for each deep edge (*w*,*r*) and its associated class H^∗^(*w*,*r*), one notes

$$\begin{aligned} &PriorState(\mathrm{H}^{\ast}(w,r),q) = AllState(w)\,\cap\\&\quad\cap \left[\bigcup\limits_{\mathrm{H} \in \mathrm{H}^{\ast}(w,r)} StartState(Back(\mathrm{H}),q) \right]\!. \end{aligned} $$

## HMM and probabilistic automata

The definition of HMM is very close to the definition of probabilistic automaton PA, [41,42]. The main difference lies in the interpretation of the behavior of a model. For a HMM, one considers a label as a symbol emitted by the HMM; for automata, one imagines an automaton that processes a given word letter by letter. Another difference connected with the previous one is that PAs are typically used to describe word sets; thus, for a given PA, the subset of accepting states is defined. HMMs are mainly used to describe probability models and thus have no accepting states.

In applications, one often uses a probabilistic automata built as a Cartesian product of a deterministic automaton accepting a given set of words and a HMM describing the word probabilities, see e.g. [6,43]. A similar construction is used below. In fact, we describe generalized probabilistic automata, GPA. As opposed to PAs, the edges in a graph that represents our automaton are labeled with words rather than with letters, and thus it can be named a generalized probabilistic automaton, analogously to the definition of generalized HMM [44].

An originality of SUFPREF is that words from pattern , or classes, that represent terminal states in classical automata need not be explicitly represented. Nevertheless, each class is uniquely associated to one deep edge.

## Probabilities equations for HMM

In the section above the main text sets and corresponding equations were described. One can apply the equations to compute probabilities of the text sets for arbitrary probability models. Here we give formulas to compute the probabilities for an HMM. The formulas are based on the following observations. First, all unions in the text equations are disjoint. Second, an item of a set union is a set with already known probability or concatenation of such sets. In the latter case the probability *Prob*(*q*_1_,*L*_1_·*L*_2_,*q*_2_) can be computed by the formula

(20) $$   Prob(q_{1}, L_{1} \cdot L_{2}, q_{2}) = \sum\limits_{\textit{\~{q}} \in Q} Prob(q_{1}, L_{1}, \textit{\~{q}})\cdot Prob(\textit{\~{q}}, L_{2}, q_{2}),  $$

where *Prob*(*q*^′^,*L*,*q*) is a probability that, being in the state *q*^′^, the chain will go to the state *q* emitting a word *t* from the set *L*.

Let *n*,*s* be integers, $w \in OV{(\mathcal {H})}$, $r \in DROV(\mathcal {H})$ and *q*∈*Q*. Then

1. From (11) follows
  (21) $$ \begin{aligned} Prob(B(n, s),q) =&\, \sum\limits_{\textit{\~{q}}\in Q}Prob(B(n-1, s),\textit{\~{q}})\cdot \pi(\textit{\~{q}},q)+ \\&+ Prob(R(n, s, \epsilon),q), \end{aligned}  $$
  where $\pi (\textit {\~{q}},q) = \sum _{a \in \mathrm {V}}\pi (\textit {\~{q}},a,q)$;
2. From (12) follows
  (22) $$ \begin{aligned} Prob(E(n, 1, r),q) =&\, \sum\limits_{\textit{\~{q}}\in StartState(\widetilde{\mathcal{H}}(r), q)} Prob(\mathrm{V}^{n-m},\textit{\~{q}}) \times\\&\times Prob(\textit{\~{q}},\widetilde{\mathcal{H}}(r),q) ; \end{aligned}  $$
3. From (13)–(15) and (17) follows
  (23) $$ \fontsize{9}{6} \begin{aligned} Prob(F(n, s + 1, r),q) =\!&\,\sum\limits_{\textit{\~{q}}\in StartState(\widetilde{\mathcal{H}}(r), q)}\!Prob(B(n - m, s),\bar{q})\times\\ &\times Prob(\textit{\~{q}},\widetilde{\mathcal{H}}(r),q); \end{aligned}  $$
  (24) $$  {\fontsize{8.9pt}{9.3pt}\selectfont{\begin{aligned} \text{Pr}\ ob(C^{\prime}(n, s \,+\, 1, w,r),q) =&\\=& \!\sum\limits_{\textit{\~{q}}\in PriorState({H}^{\ast}(w,r),q)}\!\!\!\!\!\!\!\!\!\!\!\!\!\!\!\!\!\!\!\text{Pr}\ ob(D(k(n,x),s,w),\bar{q})\times\\ &\qquad\,\,\,\,\,\times \text{Pr}\ ob(\textit{\~{q}},Back(\mathrm{H}^{\ast}(w,r)),q) ; \end{aligned}}}  $$
  (25) $$  {\fontsize{9}{6} \begin{aligned} \text{Pr}\ ob(E(n,s+1,r),q) =&\, \text{Pr}\ ob(F(n,s+1,r),q)+ \\&\!\!\!\!\!\!\!\!\!\!\!\!\!\!\!\!\!\!\!\!\!\!\!\!\!\!\!\!\!\!\!\!\!\! + \left(\sum\limits_{w:~~(w,r) \;{{is~a~deep~edge}}}\!\!\!\!\!\!\!\!\!\!\!\!\!\!\!\!\!\text{Pr}\ ob(C^{\prime}(n,s+\;1, w,r),q) {\vphantom{\sum\limits_{w:~~(w,r) \;{\text{is a deep edge}}}}}\right)\!; \end{aligned}}  $$
4. Let *lpred*(*w*)≠*ε*. Then from (10) follows
  (26) $$ {\fontsize{8.7}{6} \begin{aligned} \text{Pr}\ ob(D(k(n,w),s,w),q) =&\, \sum\limits_{\textit{\~{q}} \in PriorCloseState(w,q)} \\&\text{Pr}\ ob(D(k(n,lpred(w)), s, lpred(w)),\textit{\~{q}})\cdot \\ & \times \text{Pr}\ ob (\textit{\~{q}},Back(w),q) \\&+ \text{Pr}\ ob(R(k(n,w),s,w),q); \end{aligned}}  $$
  If *lpred*(*w*)=*ε* then
  $$\begin{array}{@{}rcl@{}} Prob(D(n,s,w),q) &=& Prob(R(n,s,w),q); \end{array} $$
5. From (5) follows
  (27) $$ \begin{aligned} Prob(RE(n, s, r),q) =&\, Prob(E(n, s,r),q)- \\&- Prob(E(n, s + 1, r),q); \end{aligned}  $$
6. Let *w* be a right deep node. Then from (6) follows
  (28) $$ {\fontsize{8.9}{6} \begin{aligned} Prob(R(n, s, w),q) =&\, Prob(RE(n, s, w),q) +\\&+ \left(\sum\limits_{x \in OV{(\mathcal{H})}: w=rpred(x)}Prob(R(n, s, x),q)\right)\!; \end{aligned}}  $$
  Otherwise, from (7) follows
  (29) $$  Prob(R(n, s, w),q) = \sum\limits_{x \in OV{(\mathcal{H})}: w=rpred(x)}Prob(R(n, s, x),q).  $$

## Algorithms

### General description

Our goal is to compute *Prob*(*B*(*N*,*S*)), that is the probability to find at least *S* occurrences of a pattern  in a random text of length *N*, given a HMM *G*=〈*Q*,*q*_0_,*π*〉. The algorithm SUFPREF, see Algorithm 1, computes the probability by induction on a text length *n*, where *m*≤*n*≤*N*, and, for a given *n*, by induction on a number of occurrences *s*, where 1≤*s*≤*S*.

The computation within the main loop is based on equations (21)–(29), related to *B*-sets, *C*-sets, *F*-sets, *E*-sets, *D*-sets, *RE*-sets and *R*-sets.

The computation related to texts of length *n* will be referred to as *n*-th stage of the algorithm’s work. The main computation within the *n*-th stage is performed by depth-first traversal of *OvGraph* following left and deep edges. During the depth-first traversal for each visited node $w \in OV{(\mathcal {H})}$, the algorithm computes the probabilities of *RE*-sets and auxiliary probabilities of *D*, *F* and *C*-sets by induction on number of occurrences *s*=1,…,*S*. Within the traversal we store the probabilities of *D*-sets related to the nodes on the path from the root of *OvGraph* to a current node *w*, i.e. the nodes *x* from *OverlapPrefix*(*w*), in the temporary arrays *TempDProb*(*x*,*q*) of the size *S*; the size of the data related to a node *x* on the path is *O*(|*Q*|×*S*), see sub-section “Main loop” below. Then update of auxiliary information stored in nodes of *OvGraph*, namely, probabilities of *R*-sets, is performed by a bottom-up traversal of *OvGraph* using right edges.

Computation on the inductive equations relies on a generic procedure, analogous to the *forward algorithm* for HMM [40], see also [5].

### Preprocessing and data structures

On the preprocessing stage we initialize the global data structures of the algorithm, i.e. the *OvGraph*, including auxiliary structures assigned to its nodes and some other structures that are described at the end of this subsection.

**Overlap graph** The graph *OvGraph* is built from the Aho-Corasick trie $T_{\mathcal {H}}$ for the set  [45]. The nodes belonging to the *OvGraph* correspond to the overlaps and therefore can be easily revealed using suffix links of the Aho-Corasick trie, see [37] and [Additional file 2], for details of the procedure. The nodes of *OvGraph* are assigned with additional data (constant data and data to be updated at each stage *n*=*m*+1,…,*N*). All these data are initialized at the preprocessing stage, see below.

**Constant transition probabilities related to nodes of overlap graph** During the computation, algorithm SUFPREF uses some probabilities that are constant and can be precomputed and stored.

- For each node *w* and all states *q* in *AllState*(*w*) and *q~* in *PriorState*(*w*,*q*), we store the “left transition probability” *Prob*(*q~*,*Back*(*w*),*q*), see definitions 15 and 16. The left transition probabilities are used for the computation of *D*-sets probabilities, see (26);
- Given a right deep node *r*, the “word probabilities” $Prob(\textit {\~{q}},\widetilde {\mathcal {H}}(r),q)$ are memorized for states *q* in *AllState*(*r*) and *q~* in *Q*. They are used to compute probabilities of the *F*-sets, see (23);
- Given a right deep node *r*, we store, for each class H^∗^(*w*,*r*), the “deep transition probabilities” *Prob*(*q~*,*Back*(H^∗^(*w*,*r*)),*q*) where *q* ranges over *AllState*(H^∗^(*w*,*r*)) and *q~* ranges over *PriorState*(H^∗^(*w*,*r*),*q*). The probabilities are needed for the computation of *C*-sets probabilities, see (24).

The sets of states *AllState*(*w*) and *PriorState*(*w*,*q*), left and deep transition probabilities and word probabilities are computed in a depth-first traversal along the left edges of *OvGraph* [see Additional file 2].

**Updatable probabilities related to nodes of overlap graph** At the beginning of the *n*-th stage, for each pair 〈*w*,*q*〉, where $w \in OV{(\mathcal {H})}$ and *q*∈*AllState*(*w*) we store a (*m*−*len*(*w*))×*S* matrix *RProbs*(*w*,*q*), where

$$RProbs(w,q)[\!i][\!s] = Prob(R(l,s,w),q); $$

*l*∈ [ *k*(*n*,*w*),*n*−1];*s*=1,…,*S*;*i*=*lmod* (*m*−*len*(*w*)). The probabilities were computed at the previous stages. And the values in the matrices are updated at the end of the *n*-th stage.

At the preprocessing stage, we compute the probabilities for *n*=1,…,*m*; *s*=1,…,*S* and *q*∈*AllState*(*w*) according to the formulas:

$$Prob(R(m,1,w),q)=Prob(\mathcal{H}(w),q); $$

if *n*<*m* or (*n*=*m* and *s*>1),

$$Prob(R(n,s,w),q)=0. $$

**The global data unrelated to overlap graph** Besides the data related to nodes of *OvGraph* we store the following data.

- Transition probabilities. For each *q~*,*q*∈*Q* we store the constant probability
  $$TransProb(\textit{\~{q}},q) = \sum_{a \in \mathrm{V}}\pi(\textit{\~{q}},a,q); $$
  At the beginning of *n*-th stage, the following values computed at the previous stages are stored
- For each *q*∈*Q*, updatable probabilities *Prob*(*V*^*n*−*m*−1^,*q*). They are used for computation of *Prob*(*E*(*n*,1,*r*),*q*) by the formula (22);
- For each *s*=1,…,*S* and *q*∈*Q*, updatable *B*-sets probabilities *Prob*(*B*(*n*−*m*−1,*s*),*q*). At the preprocessing stage, we compute the probabilities for *n*=1,…,*m*, *s*=1,…,*S* and *q*∈*Q* according to the formulas:
  $$\begin{array}{@{}rcl@{}} Prob(B(m,1),q) &=& Prob(\mathcal{H},q) ; \\ Prob(B(n,s),q) &=& 0, \;\text{if}\; n < m \\ &&\text{ or } (n=m \;\text{and}\; s> 1). \end{array} $$

### Main loop

The aim of the *n*-th stage (main loop, see lines 2–13 of the algorithm SUFPREF, see Algorithm 1) is to compute for all *s*=1,…,*S* the values

- *Prob*(*B*(*n*−*m*,*s*),*q*), *n*>2*m*;
- *Prob*(*R*(*n*,*s*,*w*),*q*) for all $ w \in OV{(\mathcal {H})}$, *q*∈*AllState*(*w*).

To compute the probabilities *Prob*(*R*(*n*,*s*,*w*),*q*) the algorithm for each pair 〈*w*,*q*〉, where $w \in OV{(\mathcal {H})}$, *q*∈*AllState*(*w*), uses local array *TempRProb*(*w*,*q*) of size *S*. Initially, for each *s*, *TempRProb*(*w*,*q*)[ *s*]=0.

The value *n* is not changed within the main loop. The body of the loop consists of three parts.

Within the part 2.1, for all *s*=1,…,*S* the values *Prob*(*B*(*n*−*m*,*s*),*q*) are computed according to the formula (21); the values *Prob*(*B*(*n*−*m*−1,*s*),*q*) and *Prob*(*R*(*n*−*m*,*s*,*ε*),*q*) were computed and stored at the previous stages.

The aim of the part 2.2 (procedure COMPUTEREPROB, see Algorithm 2) is to compute the values *Prob*(*RE*(*n*,*s*,*r*),*q*) for all $r \in DROV(\mathcal {H})$, *q*∈*AllState*(*r*) and *s*=1,…,*S*.

The computation is performed using the recursive depth-first traversal of *OvGraph* along the left edges; it is based on the formulas (22)–(27). Let a node *w* is visited, it corresponds to the call of COMPUTEREPROB (*n*,*w*). Firstly, COMPUTEREPROB computes *Prob*(*E*(*n*,1,*w*),*q*) by the formula (22) and puts the values to *TermRProb*(*w*,*q*)[ 1].

Then by induction on *s*=1,…,*S* the procedure computes the following probabilities.

Within the part B, see lines 8–14, for all states *q*∈*AllState*(*w*), the procedure computes *Prob*(*D*(*k*(*n*,*w*),*s*,*w*),*q*) by the formula (26). To make the computation by the formula (26) one needs the value *Prob*(*D*(*k*(*n*,*lpred*(*w*)),*s*,*lpred*(*w*)),*q~*); the value is stored in the array *TempDProb*(*w*,*q*), see sub-section “General description”.

Now consider the part C of Algorithm 2, see lines 15–26. Although the calculation of probabilities of *R*-sets and *RE*-sets is based on the formulas (25) and (27) we avoid explicit usage of *E*-sets in our calculations. From (25) and (27) we have (here *s*>1)

$${\fontsize{8.4}{6} \begin{aligned} \text{Pr}\,ob(RE(n, s, r), q) &= \text{Pr}\,ob(E(n, s, r), q) - \text{Pr}\,ob(E(n, s + 1, r), q) = \\ &= \text{Pr}\,ob(F(n, s, r), q)\, +\\ &+\sum\limits_{w: (w,r) \;{is~a~deep~edge}}\!\!\!\!\!\!\!\!\!\!\!\!\!\!\!\! \text{Pr}\, ob(C^{\prime}(n, s, w, r), q)\, - \\ &- \text{Pr}\,ob(F(n, s + 1, r), q) -\\ &- \sum\limits_{w: (w,r) \;{is~a~deep~edge}}\!\!\!\!\!\!\!\!\!\!\!\!\!\!\!\!\text{Pr}\,ob(C^{\prime}(n, s+1, w, r), q) = \\ &= (\text{Pr}\,ob(F(n, s, r), q) \!- \text{Pr}\,ob(F(n, s \!+ 1, r), q)) + \\ &\!\!\!\!\!\!\!\!\!\!\!\!\!\!\!\!\!\!\!\!\!\!\!\!\!\!\!\!\!\!\!\!\!\!\! +\!\! \sum\limits_{w: (w,r) \;{is~a~deep~edge}}\!\!\!\!\!\!\!\!\!\!\!\!\!\!\!\!\!(\text{Pr}\,ob(C^{\prime}(n, s, w, r), q)\! - \text{Pr}\,ob(C^{\prime}(n, s\,+\,1, w, r), q)). \end{aligned}} $$

For *s*=1 we have

$$\begin{array}{@{}rcl@{}} Prob(RE(n, 1, r), q) &=& Prob(E(n, 1, r),q) -Prob(E(n, 2, r),q) = \\ &=& Prob(E(n, 1, r),q) - Prob(F(n, 2, r), q) \\&&- \sum\limits_{w: (w,r) \;\text{is a deep edge}}Prob(C^{\prime}(n, 2, w, r), q). \end{array} $$

The value *Prob*(*E*(*n*,1,*r*),*q*) was computed and stored in *TempRProb*(*w*,*q*)[ 1] at the part A of the procedure. During the computation we accumulate the needed probabilities in the arrays *TempRProb*(*w*,*q*), see section C of the algorithm 2, lines 15–26. Visiting a left deep node *w*, for each *r* such that there is a deep edge (*w*,*r*), and for each *q*∈*AllState*(*r*), we firstly calculate the value *Prob*(*C*^′^(*n*,*s*+1,*w*,*r*),*q*) using (24). Then add to the current value of *TempRProb*(*w*,*q*)[ *s*] the value *Prob*(*C*^′^(*n*,*s*,*w*,*r*),*q*)−*Prob*(*C*^′^(*n*,*s*+1,*w*,*r*),*q*) (if *s*>1) or substract the value *Prob*(*C*^′^(*n*,2,*w*,*r*),*q*) (if *s*=1).

In section D of the Algorithm 2, see lines 27–36 we analogously take into account the probabilities of *F*-sets.

At part 2.3 of the algorithm SUFPREF (procedure COMPUTERPROB, see Algorithm 3), the values *Prob*(*R*(*n*,*s*,*w*),*q*) are computed according to the formulas (28), (29).

The computation is done by a recursive bottom-up traversal of *OvGraph* along the right edges. Also the procedure records the computed *Prob*(*R*(*n*,*s*,*w*),*q*) probabilities to the corresponding cells of the matrix *RProb*(*w*,*q*) and initializes elements of *TempRProb*(*w*,*q*) by zeros.

#### **Remark**.

The above traversals are implemented with a recursive procedure initially called at the root (node corresponding to *ε*) of *OvGraph*, see lines 11, 12 of the algorithm SUFPREF (Algorithm 1).

### Post-processing

At the post-processing step of the algorithm (see Algorithm 1, lines 14–19), *P*-value *Prob*(*B*(*N*,*S*)) follows by summation over *Q* states:

$$Prob(B(N,S)) = \sum\limits_{q \in Q} Prob(B(N,S),q) ~~. $$

## Discussion

**Space complexity** The data stored consist of input data, temporary data used at the preprocessing step, the main data structure *OvGraph* and the working data unrelated to the *OvGraph*. The detailed description of all of the data is given in the section “Preprocessing and data structures”. The space complexity is mainly determined by the memory needed for the data related to the *OvGraph* and temporary data used at the preprocessing step. We first briefly consider the data unrelated to the overlap graph; then we consider *OvGraph* data. The input data consist of the text length *N*, the number of occurrences *S*, a representation of an HMM and a pattern . The data related to the pattern representation are included in the data related to *OvGraph* nodes and will be considered below. Storage size for an HMM is *O*(|*Q*|^2^×|V|). Thus the input data size is *O*(|*Q*|^2^×|V|).

At the preprocessing stage the algorithm uses a temporary structure, the Aho-Corasick trie, to build the *OvGraph* and temporary data structures to store intermediate probabilities *Prob*(*q~*,*Back*(*w*),*q*) for each $w \in OV{(\mathcal {H})}$, and probabilities *Prob*(*q~*,*Back*(H),*q*) and *Prob*(*q~*,H,*q*) for each $\mathrm {H} \in \mathcal {H}$, where *q~*,*q*∈*Q*. The memory needed for Aho-Corasick trie is $O(m \times |\mathcal {H}|)$ where *m* is the pattern length. The memory needed to store the intermediate probabilities is $O(|Q|^{2} \times (|OV{(\mathcal {H})}| + |\mathcal {H}|))$. The temporary data structures used by sub-algorithms in the preprocessing stage are released after their running. Thus, the total memory used during this stage is $O(|Q|^{2} \times (|OV{(\mathcal {H})}| + |\mathcal {H}|) + m \times |\mathcal {H}|)$.

The working data unrelated to *OvGraph* consist of *B*-sets probabilities *Prob*(*B*(*n*−*m*−1,*s*),*q*) and probabilities *Prob*(*V*^*n*−*m*−1^,*q*), *q*∈*Q*. These data need *O*(|*Q*|×*S*) and *O*(|*Q*|) memory, respectively. Within the main loop we use local arrays with *D*-sets probabilities (the number of these arrays is at most *m*×|*Q*|, see remark below) and arrays *TempRProb*(*w*,*q*) (for all $w \in OV{(\mathcal {H})}$, *q*∈*AllState*(*w*)). These arrays are of size *S*. Therefore the necessary memory to store all of the arrays is $O(|Q|\!\times \! S \!\times \! m \,+\, |Q| \!\times \! S\!\times \!| OV{(\mathcal {H})}|)$.

As we will see, all this memory, except for the memory needed to store Aho-Corasick trie, does not increase the space complexity of the algorithm.

### **Remark**.

During processing of a node *w* in main loop one stores arrays with *D*-set probabilities for all left predecessors of *w*, i. e. for all *x*∈*OverlapPrefix*(*x*). The number of left predecessors is bounded by the number of all prefixes of *w*, that is *len*(*w*), where *len*(*w*)≤*m*. Thus the number of arrays with *D*-sets probabilities used by the algorithm during the performing of main loop is at most *m*×|*Q*|.

Consider now the data related to the *OvGraph*. The *OvGraph* structure is determined by the pattern . The number of nodes and the number of left and right edges is $O(|OV{(\mathcal {H})}|)$, that is upper bounded by $m \times |\mathcal {H}|$. However, usually $|OV{(\mathcal {H})}| \leq |\mathcal {H}|$, see Table 1. The number of deep edges is equal to the number of classes, $|\mathcal {P}(\mathcal {H})|$, that is upper bounded by $|\mathcal {H}|$. Then the storage size for *OvGraph* is $O(|OV{(\mathcal {H})}| + |\mathcal {H}|)$. The data assigned to a node of *OvGraph* consist of constant data and updatable data. The constant data consist of left transition probabilities assigned to the nodes of the *OvGraph*, deep transition probabilities assigned to the deep edges and word probabilities assigned to the right deep nodes. The updatable data are probabilities of *R*-sets assigned to all nodes. More precisely, left transition probabilities *Prob*(*q~*,*Back*(*w*),*q*) are stored in the memory associated with a node *w*; deep transition probabilities *Prob*(*q~*,*Back*(H^∗^(*w*,*r*)),*q*) are stored in the memory associated with deep edge (*w*,*r*); word probabilities $Prob(\textit {\~{q}}, \widetilde {\mathcal {H}}(r),q)$ are stored in the memory associated with a right deep node *r*. As a whole, it gives

$$\begin{aligned} &O(|Q|^{2} \times |OV{({H})}|) + O(|Q|^{2} \times |{P}({H})|)\, +\\ &+ O(|Q|^{2} \times |DROV({H})|)\leq\\ & \leq O(|Q|^{2} \times (|OV{({H})}| + |{H}|)). \end{aligned} $$

**Table 1:** **PSSM-based patterns of length 12**

**Pattern **	${Fraction(\boldsymbol{\mathcal {H}})}$		${OV(\boldsymbol{\mathcal {H}})}$	${\boldsymbol{\mathcal {P}}(\boldsymbol{\mathcal {H}})}$	***N***_***AC***_	***P*** **-value**
PSSM(12,9.63)	0.00001	169	14	57	468	2.1887831E-27
PSSM(12,8.69)	0.00003	503	22	125	1123	9.9588634E-22
PSSM(12,7.41)	0.0001	1682	49	395	3189	2.1630650E-16
PSSM(12,5.89)	0.0003	5045	157	1789	9070	3.9649240E-12
PSSM(12,4.01)	0.001	16835	488	8967	29297	2.0930535E-07
PSSM(12,2.04)	0.003	50490	1417	35313	83016	0.001494591

The number *x* in “PSSM(12,*x*)” denotes the cut-off. The *P*-values are given w.r.t. the text length and probability models described in the text of the paper. The intermediate values of $\textit {Fraction}(\mathcal {H})$ (0.003, 0.0003, etc. instead of more common 0.005, 0.0005, etc.) were chosen to obtain more homogeneous log-scale.

To store *R*-sets probabilities one needs $O(S \times |Q| \times m \times |OV{(\mathcal {H})}|)$ memory. Thus the size of memory needed to store global data related to *OvGraph* is

$$O(|Q|^{2} \times (|OV{(\mathcal{H})}|+|\mathcal{H}|)+ |Q| \times S \times m \times |OV{(\mathcal{H})}|). $$

Finally, the overall space complexity of the algorithm is

$${} \begin{aligned} &O(|Q|^{2} \times (|OV{(\mathcal{H})}|+|\mathcal{H}|)+ |Q| \times S \times m \times |OV{(\mathcal{H})}|+\\&\quad+m \times |\mathcal{H}|). \end{aligned} $$

Observe that the storage of classes in deep nodes saves a $O(S\times |Q| \times m \times |\mathcal {P}(\mathcal {H})|)$ memory for *R*-sets.

### **Remark**.

The parameter $|OV(\mathcal {H})|$ belongs to the bounds of space and time complexities. It is upper bounded by $ m \times |\mathcal {H}|$. Assume that a pattern consists of random words of length *m* generated according to the uniform Bernoulli model. It was shown that in such case $|OV(\mathcal {H})| \approx |\mathcal {H}|$, see [46] and supplementary materials, file “Comparison_with_AhoPro.xls”. But for a majority of patterns described by Position-Specific Scoring Matrices and cut-offs that were considered in the present paper, $|OV(\mathcal {H})| \leq 0.1 \times |\mathcal {H}|$, see Table 1 in this paper and [Additional file 3].

**Time complexity** The algorithm SUFPREF (see Algorithm 1) consists of three parts: preprocessing, main loop and post-processing. The time complexity of the pre-processing part is mainly determined by the construction of the Aho-Corasick trie and *OvGraph*, their traversals and the computation of intermediate probabilities. The complexity is $O(|Q|^{2} \times m \times (|OV{(\mathcal {H})}| + |\mathcal {H}|)$. Some details are given in [Additional file 2]. The time complexity of the post-processing part (see lines 14–19) is *O*(*m*×|*Q*|^2^).

The time complexity of the algorithm SUFPREF is mainly determined by the main loop (see lines 2–13), i.e. by the total run-time of the computation of parts 2.1, 2.2 and 2.3 for *n*=*m*+1,…,*N*. Within the part 2.1 (lines 3–10), computing probabilities *Prob*(*B*(*n*−*m*,*s*),*q*) for all *s*=1,…,*S* and *q*∈*Q* requires *O*(*S*×|*Q*|^2^) operations.

Consider the part 2.2 (procedure COMPUTEREPROB, see Algorithm 2). The procedure performs computations by depth-first traversal of *OvGraph* for all $w \in OV{(\mathcal {H})}$. For a given *n* and *w* the computation consists of four parts: A, B, C and D. If *w* is right deep node then at the part A (lines 1–6) one computes *Prob*(*E*(*n*,1,*w*),*q*) for all *q*∈*AllState*(*w*); overall nodes this requires $O(|Q|^{2} \times |OV{(\mathcal {H})}|)$ operations.

The parts B, C and D run for *S* values of *s*. To execute parts B, C and D (lines 8–14, 15–26 and 27–36 respectively) overall nodes of *OvGraph* one needs $O(S \times |Q|^{2} \times |OV{(\mathcal {H})}|)$, $O(S \times |Q|^{2} \times (|OV{(\mathcal {H})}| + |\mathcal {H}|))$ and $O(S \times |Q|^{2} \times |OV{(\mathcal {H})}|)$ operations respectively.

As a whole, $O(S \times |Q|^{2} \times (|OV{(\mathcal {H})}| + |\mathcal {H}|))$ operations are needed to execute COMPUTEREPROB.

Analogously, for computation of part 2.3 (see procedure COMPUTERPROB, see Algorithm 3) one needs $O(|Q| \times S \times |OV{(\mathcal {H})}|)$ operations. Therefore, the time complexity of the algorithm SUFPREF for a general HMM is

$$O(N \times S \times |Q|^{2} \times(|OV{(\mathcal{H})}| + |\mathcal{H}|)). $$

**Time and space asymptotics** In the previous sub-section we gave upper bounds of the space and time complexities of the algorithm SUFPREF. All bounds are given as big-O notations. For example, the time complexity bounds have form $N \times S \times \lambda (G)\times \mu (\mathcal {H})$, here *N* is the text length, *S* is the number of occurrences, *λ*(*G*) is a factor depending on the HMM *G* and $\mu (\mathcal {H})$ is a factor depending on the pattern . The estimation of space complexity is analogous except of absence of factor *N*, see sub-section “Space complexity” for details.

In the case of a general HMM *λ*(*G*)=*k*×|*Q*|^2^, here |*Q*| is the number of states of the HMM *G*; the value of *k* depends on features of the HMM.

We have performed computer experiments to get a better understanding of the asymptotic behavior of time and space complexity. Let *N*_*Trans*_ be the number of states where the HMM can transit in one step from a given state. This parameter describes the “density” of an HMM; the smaller *N*_*Trans*_, the smaller the complexities of the algorithm. The factor *λ*(*G*) was studied as a function of *N*_*Trans*_ and the number of states *N*_*States*_ in used HMMs. We have performed 96=4×24 series of experiments, 100 experiments in each series. In all series we have used following input data:

- the pattern is defined by a PSSM for transcription factor FOXA2 from the database HOCOMOCO [47] and cut-off 5.89 that corresponds to roughly 0.03*%* of all words of length 12;
- number of occurrences is 10;
- text length is 1000.

**Figure 2:**
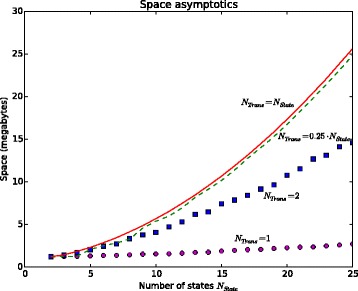
**Average size of used memory of** SUFPREF **.** The details of the experiments are given in [Additional file 4]. The computer environment is described in the subsection “Comparison with the existing algorithms”.

**Figure 3:**
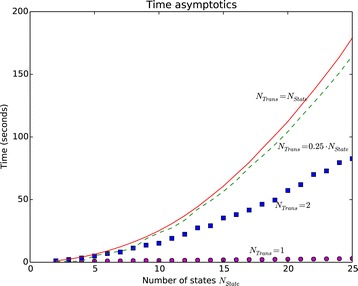
**Average run-time of** SUFPREF **.** The details of the experiments are given in [Additional file 4]. The computer environment is described in the subsection “Comparison with the existing algorithms”.

Thus, a series differs from the others only with the used HMMs. Each series is determined by the number *N*_*State*_ of states in the HMMs, and the number *N*_*Trans*_, see above. The value *N*_*State*_ ranges from 2 to 25, therefore 24 values of *N*_*State*_ were considered. For each number of states four values of *N*_*Trans*_ were used, namely, 1;2;0.25·*N*_*State*_ and *N*_*State*_. Given values *N*_*State*_ and *N*_*Trans*_, we have created 100 HMMs by the following randomized procedure. For each state *q~*, we firstly have randomly chosen *N*_*Trans*_ states *q*∈*Q* such that there exists a transition from *q~* to *q*. In our models if there exists transition from *q~* to *q* then *π*(*q~*,*a*,*q*)>0 for all *a*∈V. Then we assign to each triple 〈*q~*,*a*,*q*〉, where *a*∈V, a random positive value *π*(*q~*,*a*,*q*) from [0,1], and then normalize the values to make the needed sums of probabilities equal to 1. For each series we report average run-time and used space. The results of the experiments are presented in Figure 2 and Figure 3. The experiments show that for *N*_*Trans*_=0.25·*N*_*State*_ and *N*_*Trans*_=*N*_*State*_ the time and space are not much different. This is because most of states from *Q* are reachable for nodes of overlap graph. In contrast, for *N*_*Trans*_=1 (the models are deterministic) the run-time and used space are significantly less than in the case considered above. The case *N*_*Trans*_=2 is an intermediate case. Note that Markov models are deterministic and correspond to the case *N*_*Trans*_=1.

In the cases *N*_*Trans*_=2;0.25·*N*_*State*_ and *N*_*State*_, a proportion *k*×|*Q*|^2^ is reached. The smaller is *N*_*Trans*_, the smaller is *k*. When *N*_*Trans*_=1, the function *λ*(*G*) has approximately linear behavior.

Analogous experiments for patterns described by other PSSMs and cut-offs show the same results. The results are given in [Additional file 4].

Now let’s consider in details the complexity of the algorithm for Bernoulli and Markov models.

**Bernoulli models** In a Bernoulli model, the set *Q* contains only 1 state. Therefore formulas for space and time complexities turn into $O(m \times S \times |OV{(\mathcal {H})}|+ m \times |\mathcal {H}|)$ and $O(N \times S \times (|OV{(\mathcal {H})}| + |\mathcal {H}|))$. Note (see algorithm SUFPREF, Algorithm 1) that time and space complexity of the algorithm does not depend on symbol probabilities given by a Bernoulli model [see Additional file 5].

**Markov models. Further refinements** Complexity results are presented with (possibly rough) upper bounds. In particular, the |*Q*|^2^ factor arises from transition probabilities representation. It actually stands for the sum of the cardinalities of *PriorState*(*w*,*q*) sets in a given node $w \in OV{(\mathcal {H})}$, *q*∈*AllState*(*w*).

In practical cases, this number may be significantly smaller than |*Q*|^2^. In particular, this is the case for Markov models that can be treated as a special case of Hidden Markov Models. Let *K* denote the order of Markov model. For an overlap node *w*, such that *len*(*w*)≥*K*, the set *AllState*(*w*) consists of only one state. We use the technique of “reachable states”, see section “Probability models” to take into account this issue. It does not decrease the upper bounds in the case of a general HMM but leads to a significant improvement of the software for Markov models. At the same time, combining the technique with other improvements of the algorithm, see [37], allows one to obtain better complexity bounds for the Markov case. Namely, $O(S \times m \times (K \times |\mathrm {V}|^{K+1}+|OV{(\mathcal {H})}|)+m \times |\mathcal {H}|)$ space complexity and $O(N \times S \times (K \times |\mathrm {V}|^{K+1} +|OV{(\mathcal {H})}|+|\mathcal {H}|))$ time complexities are achievable. The details of the optimized algorithm for the Markov case achieving the above bounds will be presented in a separate paper.

**Comparison with the existing algorithms** The algorithm SUFPREF implements a *P*-value computation for HMM and achieves the theoretical complexity of the best algorithms for *P*-value computation. Notably, the complexities of SUFPREF are consistent with the complexities of algorithms based on finite automata. Our optimization of the data structure provides an improvement for the constant factor. A comparison of the number of nodes of *OvGraph* and the number of states of a minimal automaton for a given pattern is given in paper [37]. It was observed in [46] that an average number of overlaps (nodes of *OvGraph*) for random patterns generated according to Bernoulli models is proportional to the number of words in the patterns and is independent of the length of the words.

For Bernoulli and first order Markov model cases, we have compared program SUFPREF with the implementation of program AHOPRO [31]. The program AHOPRO admits only Bernoulli and first order Markov models.

The *P*-values were computed with the following input parameters: (1) alphabet (V) - {A, C, G, T}; (2) Bernoulli probabilities of letters - {0.25,0.25,0.25,0.25}; Markov model is described by a 4×4 matrix where all elements are 0.25; (3) text length - 1000; (4) minimal number of occurrences - 10 and (5) two types of patterns: patterns containing words of lengths 12 and 14 randomly generated according to a uniform probability model and patterns of lengths 12 and 14 defined by a Position-Specific Scoring Matrix (PSSM or PWM) and different cut-offs. A pattern presented by PSSM and cut-off consists of all words whose score according to PSSM is greater than the cut-off. The cut-offs were precalculated such that the numbers of words matching the PSSM and a cut-off are equal to the fractions of all 12 (14)-mers in range from 0.00001 to 0.003. The fractions correspond to the fractions of words in patterns using for transcription factor binding sites (TFBS) prediction.

The experiments were performed using a quad-core Intel Core i5 system running at 2.67 GHz (only one core used) with 8 GB RAM and dual-disk stripped swap partition. Both programs AHOPRO and SUFPREF were compiled using the GCC 4.5 tool chain for the 64-bit Linux target. To measure running time and maximum sizes of memory during the program’s runs we used POSIX’s “getrusage()” function twice: before and after processing to measure data size excluding program code itself. We have slightly modified the source code of AHOPRO to call this function before and after main program execution.

We consider the matrices PSSM from the database HOCOMOCO [47] describing binding sites of lengths 12 and 14 in human genome for transcription factors FOXA2 and E2F1; the matrices are given in [Additional file 6]. Observe that the *P*-values computed for both probability models are the same, when the other parameters are identical.

The results of the experiments for PSSM-based patterns of length 12 are presented in Tables 1 and 2. The results for other patterns are given in [Additional file 7]. Table 1 provides details on the patterns structures; *N*_*AC*_ denotes the number of nodes of the Aho-Corasick trie (the size of automaton used by AHOPRO). Table 2 provides space and run-time results. The running time is given in seconds and the memory size in megabytes.

**Table 2:** **Comparison of running time and used space of** SUFPREF **and** AHOPRO **programs for PSSM-based patterns of length 12**

**Experiments parameters**			**Time**			**Space**		
**Pattern **	${Fraction(\boldsymbol{\mathcal {H}})}$	**Prob Distrib**	**SufPref**	**AhoPro**	**Aho/SP**	**SufPref**	**AhoPro**	**Aho/SP**
PSSM(12,9.63)	0.00001	Bernoulli	0.02	0.37	20.39	0.44	0.59	1.36
PSSM(12,8.69)	0.00003	Bernoulli	0.03	0.90	32.00	0.5	0.97	1.94
PSSM(12,7.41)	0.0001	Bernoulli	0.07	2.60	37.64	0.69	1.88	2.74
PSSM(12,5.89)	0.0003	Bernoulli	0.27	7.64	28.10	1.21	4.97	4.11
PSSM(12,4.01)	0.001	Bernoulli	1.27	26.15	20.61	3.01	15.28	5.07
PSSM(12,2.04)	0.003	Bernoulli	4.99	78.37	15.70	7.75	42.61	5.50
PSSM(12,9.63)	0.00001	Markov	0.03	0.38	15.12	0.47	0.62	1.32
PSSM(12,8.69)	0.00003	Markov	0.05	0.91	18.65	0.53	0.97	1.84
PSSM(12,7.41)	0.0001	Markov	0.11	2.64	23.13	0.71	1.91	2.67
PSSM(12,5.89)	0.0003	Markov	0.41	7.74	18.78	1.24	5.02	4.04
PSSM(12,4.01)	0.001	Markov	1.77	26.50	14.95	3.04	15.31	5.04
PSSM(12,2.04)	0.003	Markov	6.67	79.25	11.88	8.36	42.65	4.94

See Table 1 for the general information on the patterns. The intermediate values of $\textit {Fraction}\boldsymbol {(}\mathcal {H}\boldsymbol {)}$ (0.003, 0.0003, etc. instead of more common 0.005, 0.0005, etc.) were chosen to obtain more homogeneous log-scale.

It turns out that in all cases our program is several times faster than AHOPRO. And for a majority of cases, it is faster than AHOPRO by more than ten and five times for Bernoulli and Markov models respectively. Also it outperforms AHOPRO in space.

### **Remark**.

The advantages of SUFPREF are crucial for patterns of big sizes. For example, consider the pattern described by PSSM corresponding to binding sites of lengths 16 for factor ANDR with cut-off 4.64, where pattern contain about 0.001 of all 16-mers (4270349 words). For this pattern, the run time and space of SUFPREF’s work are 12.71 seconds and 691.58 megabytes. But the run time and space of AHOPRO’s work are 351.59 seconds and 1868.18 megabytes.

### **Remark**.

For a Bernoulli model the time complexities of AHOPRO and SUFPREF are *O*(*N*×*S*×|V|×*N*_*AC*_) and $O(N \times S \times (|OV{(\mathcal {H})}| + |\mathcal {H}|))$. Note, $N_{\textit {AC}} \geq |OV{(\mathcal {H})}| + |\mathcal {H}|$.

**Usage of*P*-values for TFBS prediction** The majority of methods for TFBS prediction firstly search for genome regions with high number of occurrences of a pattern corresponding to needed TFBS. Then the candidate regions have to be chosen following proper criterion of statistical significance [48,49]. We have compared predictive abilities of methods using criteria based on *P*-values for different probability models and a method using criterion based on a number of occurrences. The experiments were performed with human transcription factor FOXA2. We have considered several patterns based on the PSSM of length 12 from the database HOCOMOCO [47] and different cut-offs. The best results were obtained for the cut-off 5.89; about 0.0003 of all words of length 12 match the PSSM with this cut-off. The pattern  that is discussed below consists of all words having score exceeding the cut-off and their reverse-complemented words.

We have considered the test set of 1800 genome regions of length from 200 to 400; the set consists of 900 “positive” regions and 900 “negative” ones. The positive regions were taken from the database ENCODE [50]. We have chosen top 900 regions related to human transcription factor FOXA2 having length from 200 to 400 b.p. in accordance with their quality (Signal value). The length distribution of regions is almost uniform; all the regions belong to Top 1000 of the FOXA2-related regions according to their Signal value. The negative regions presumably do not bind FOXA2. They were taken from random places of the first chromosome of human genome, the length of negative regions by construction are uniformly distributed from 200 to 400 b.p. For each region (positive or negative) we have computed 5 variants of *P*-values related to different probability models. The other parameters of computation were chosen as follows.

1. Text length *N* is the length of the region.
2. Number of pattern occurrences *S* is the number of occurrences of the pattern found in the region.
3. Let *MinScore* be the minimal PSSM score among scores of the pattern words found in the region. The pattern $\mathcal {H}^{\prime }$ used within the *P*-value calculation corresponds to the FOXA2 PSSM and the cut-off *MinScore*.

The *P*-values were calculated w.r.t. five probability models (for each model it’s short notation is given): Bernoulli (Bernoulli), Markov models of orders 1 (Markov1) and 2 (Markov2), HMM with 3 states (HMM3) and 4 states (HMM4). The parameters of the models were estimated on the adjacent fragments of length 4000 b.p. taken from both sides of the considered region. To estimate parameters of Bernoulli and Markov models we have used maximal likelihood method; for HMMs we have used Baum-Welch algorithm, see [40].

**Figure 4:**
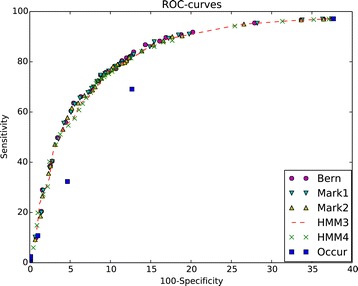
**ROC-curves for recognition methods.** The methods are described in the text and Table 3. Blue squares correspond to the method based on the number of occurrences. The ROC-curves for *P*-value based methods are almost coincide.

The main results are given in Table 3 and Figure 4; the details of the experiments are given in [Additional file 8]. The Table shows sensitivity and specificity of recognition for various thresholds and probability models. The thresholds for *P*-value based methods were chosen to obtain approximately the same sensitivity as the method based on number of occurrences with corresponding minimal number of occurrences. One can see (see also Figure 4) that all *P*-value methods have approximately the same quality and outperform the method based on number of occurrences.

**Table 3:** **Sensitivity and specificity of TFBS recognition for various thresholds and probability models**

	**Number of occurrences**	***P*** **-value**				
		**Bernoulli**	**Markov1**	**Markov2**	**HMM3**	**HMM4**
Threshold	1	0.5	0.5	0.5	0.5	0.8
Sensitivity	97.11%	97.11%	97.11%	97.11%	97.11%	97.11%
Specificity	62.33%	62.56%	62.56%	62.56%	62.78 %	62.33%
Threshold	2	0.0189	0.01966	0.0215	0.0232	0.02619
Sensitivity	69.11%	69.11%	69.11%	69.11%	69.11%	69.22%
Specificity	87.33%	92.33%	92.33%	92%	92%	92.22%
Threshold	3	0.00135	0.00135	0.00157	0.00219	0.003
Sensitivity	32.33%	32.44%	32.44%	32.44%	32.44%	32.33%
Specificity	95.33%	98.11%	98%	98%	97.56%	97.78%

See details in the text of the paper.

### **Remark**.

The signal value of ChIP-Seq data reflects the amount of binded proteins. Therefore the signal values of considered ENCODE regions show better correlation with number of pattern occurrences, than with *P*-values, see Table 4. However, the methods for TFBS prediction based on *P*-values show significantly better predictive abilities.

**Table 4:** **Spearman’s rank correlation between experimental ENCODE signal value and characteristics of regions related to pattern occurrences**

	**Number of occurrences**	***P*** **-value**				
		**Bernoulli**	**Markov1**	**Markov2**	**HMM3**	**HMM4**
Spearman’s coef.	0.12	0.061	0.061	0.058	0.059	0.063
Significance level	0.0003	0.0674	0.0673	0.0802	0.0796	0.0578

See the text for further explanations.

## Conclusions

This work presents an approach to compute the *P*-value of multiple pattern occurrence within a randomly generated text of a given length. The approach provides significant space and time improvements compared to the existing software that is crucially important for applications. The improvements are achieved due to the use of an overlap graph: taking into account overlaps between the pattern words allows one to decrease necessary space and time. The number of nodes of a Aho-Corasick trie, a structure that is extensively used in automaton approach, is much larger than the number of overlaps.

Another advantage is that, unlike existing algorithms and programs, it allows us to deal with Hidden Markov Models, the most general class of popular probabilistic models. The algorithm relies on the Cartesian product of the overlap graph and the graph of HMM states. A further reduction to the reachable vertices leads to extra improvement of time and space complexity. Despite the fact that Bernoulli and Markov models can be treated as special HMMs, it is worth implementing specialized and optimized versions of software for these models. Indeed, paper [37] can be viewed as a meta version of SUFPREF. The peculiarity of the implementation of Markov models of higher orders will be presented in a separate paper.

The implementation of the algorithm SUFPREF was compared with the program AHOPRO for a Bernoulli model and a first order Markov model. The comparison shows that, for a majority of cases, our algorithm is faster than AHOPRO in more than ten times for the Bernoulli model and in more than five times for the Markov model. The greatest advantage of SUFPREF is to decrease the needs in space. It outperforms AHOPRO in space. Therefore it can be run with patterns with a greater number of words and a greater length.

## Availability and requirements

The algorithm SUFPREF was implemented as a C++ program and was compiled for Unix and Windows. The program was implemented both as web-server and as a standalone program with the command line interface. It is available at http://server2.lpm.org.ru/bio/online/sf/. Implementation details are provided in http://server2.lpm.org.ru/static/downloads/SufPrefHMM/Web-site.pdf.

The algorithm SufPref supports *P*-values computation taking into account pattern occurrences on the both strands of genome fragments. To do this the algorithm adds to the pattern reverse complement words to the words from the pattern. After the procedure, the pattern size is not increased by more than twice.

## Additional files

Additional file 1
**Proofs of propositions.** Description of data: The file contains the proofs of the propositions 2 and 3.

Additional file 2
**Algorithms of pre-processing stage of** SUFPREF **.** Description of data: The file contains description of the algorithms used on the pre-processing stage of the algorithm SufPref.

Additional file 3
**Number of overlaps between pattern words.** Description of data: The file contains information about numbers of overlaps between words of the patterns defined by PSSMs from HOCOMOCO [47]. The detailed description of the data is given in sheet “INFO” of the file.

Additional file 4
**Time and space asymptotics.** Description of data: The file contains the details of experiments that were performed for understanding of the asymptotic behavior of time and space complexities of the algorithm SufPref for HMMs. The detailed description of the data is given in sheet “INFO” of the file.

Additional file 5
**Running time and used space of** SUFPREF **for Bernoulli models.** Description of data: The file contains results of comparison of running time and used space of SufPref’s work for several Bernoulli models.

Additional file 6
**PSSMs from HOCOMOCO related to transcription factors ANDR, ATF1, E2F1 and FOXA2.**

Additional file 7
**Comparison of** SUFPREF and AHOPRO **.** Description of data: The file contains the details of comparison of running time and used space of the programs SufPref and AhoPro. The detailed description of the data is given in sheet “INFO” of the file.

Additional file 8
**Accuracies of TFBS prediction methods.** Description of data: The file contains the details of comparison of methods for TFBS prediction based on *P*-values and on number of occurrences. The detailed description of the data is given in sheet “INFO” of the file.

## Abbreviations

HMM: Hidden Markov model
PSSM: Position-specific scoring matrix synonym of PWM;
PWM: Position weight matrix
TFBS: Transcription factor binding sites

## References

[1] Qian Z, Lu L, Qi L, Li Y (2007). **An efficient method for statistical significance calculation of transcription factor binding sites**. Bioinformation.

[2] Berman B, Pfeiffer B, Laverty T, Salzberg S, Rubin G, Eisen M, Celniker S (2004). **Computational identification of developmental enhancers: conservation and function of transcription factor binding-site clusters in Drosophila melanogaster and Drosophila pseudoobscura**. Genome Biol.

[3] Cartharius K, Frech K, Grote K, Klocke B, Haltmeier M, Klingenhoff A, Frisch M, Bayerlein M, Werner T (2005). **MatInspector and beyond: promoter analysis based on transcription factor binding sites**. Bioinformatics.

[4] Helden JV, Olmo M, Perez-Ortin J (2000). **Statistical analysis of yeast genomic downstream sequences revels putative polyadenylation signals**. Nucleic Acids Res.

[5] Roytberg MA (2009). **Computation of the probabilities of families of biological sequences**. Biophysics.

[6] Marschal T, Herms I, Kaltenbach H, Rahmann S (2012). **Probabilistic arithmetic automata and their applications**. IEEE/ACM Trans Comput Biol Bioinformatics.

[7] Reinert G, Schbath S (2000). **Probabilistic and statistical properties of words: an overview**. J Comput Biol.

[8] Tompa M, Li N, Bailey T, Church G, De Moor B, Eskin E, Favorov A, Frith M, Fu Y, Kent J, Makeev V, Mironov A, Noble W, Pavesi G, Pesole G, Régnier M, Simonis N, Sinha S, Thijs G, van Helden J, Vandenbogaert M, Weng Z, Workman C, Ye C, Zhu Z (2005). **An assessment of computational tools for the discovery of transcription factor binding sites**. Nat Biotechnol.

[9] Nuel G (2006). **Numerical solutions for patterns statistics on Markov chains**. Stat Appl Genet Mol Biol.

[10] Lladser M, Betterton MD, Knight R (2008). **Multiple pattern matching: A Markov chain approach**. J Math Biol.

[11] Guibas L, Odlyzko A (1981). **String overlaps, pattern matching and nontransitive games**. J Comb Theory, Series A.

[12] Szpankowski W (2001). *Average case analysis of algorithms on sequences*.

[13] Régnier M (2000). **A unified approach to word occurrences probabilities**. Discrete Appl Math.

[14] Régnier M, Szpankowski W (1997). **On pattern frequency occurrences in a Markovian sequence**. Algorithmica.

[15] Régnier M, Denise A (2004). **Rare events and conditional events on random strings**. Discrete Math Theor Comput Sci.

[16] Nicodéme P (2004). **Motif statistics**. Theor Comput Sci.

[17] Nicodéme P (2003). **Regexpcount, a symbolic package for counting problems on regular expressions and words**. Fundamenta Informaticae.

[18] Régnier M, Lifanov A, Makeev V: **Three variations on word counting**. In *Proceedings German Conference on Bioinformatics*. Heidelberg; 2000:75–82.

[19] Prum B, Rodolphe F, Turckheim E (1995). **Finding words with unexpected frequencies in DNA sequences**. J R Stat Soc B.

[20] Bender EA, Kochman F (1993). **The distribution of subword counts is usually normal**. Eur J Comb.

[21] Cowan R (1991). **Expected frequencies of DNA patterns using Whittle’s formula**. J Appl Prob.

[22] Godbole AP (1991). **Poissons approximations for runs and patterns of rare events**. Adv Appl Prob.

[23] Geske MX, Godbole AP, Schaffner AA, Skrolnick AM, Wallstrom GL (1995). **Compound Poisson approximations for word patterns under Markovian hypotheses**. J Appl Prob.

[24] Reinert G, Schbath S (1998). **Compound Poisson approximation for occurrences of multiple words in Markov chains**. J Comput Biol.

[25] Nuel G (2008). **Pattern Markov chains: optimal Markov chain embedding through deterministic finite automata**. J Appl Prob.

[26] MR L, Spouge J, Kanga G, Landsman D (2004). **Statistical analysis of over-represented words in human promoter sequences**. Nucleic Acids Res.

[27] Regnier M, Vandenbogaert M (2006). **Comparison of statistical significance criteria**. J Bioinformatics Comput Biol.

[28] Regnier M, Bourdon J (2014). **Large deviation properties for patterns**. J Discrete Algorithms.

[29] Nuel G (2004). **LD-SPatt: large deviations statistics for patterns on Markov chains**. J Comp Biol.

[30] Hertzberg L, Zuk O, Getz G, Domany E (2005). **Finding motifs in promoter regions**. J Comput Biol.

[31] Boeva V, Clément J, Régnier M, Roytberg M, Makeev V (2007). **Exact p-value calculation for heterotypic clusters of regulatory motifs and its application in computational annotation of cis-regulatory modules**. Algorithms Mol Biol.

[32] Nuel G (2006). **Effective p-value computations using finite Markov chain imbedding (FMCI): application to local score and to pattern statistics**. Algorithms Mol Biol.

[33] Zhang J, Jiang B, Li M, Tromp J, Zhang X, Zhang M (2006). **Computing exact p-values for DNA motifs**. Bioinformatics.

[34] Fu J, Lou W (2003). *Distribution theory of runs and patterns and its applications. A finite Markov chain imbedding approach*.

[35] Crochemore M, Stefanov V (2003). **Waiting time and complexity for matching patterns with automata**. Inform Process Lett.

[36] Ribeca P, Raineri E (2008). **Faster exact Markovian probability functions for motif occurrences: a DFA-only approach**. Bioinformatics.

[37] Regnier M, Kirakossian Z, Furletova E, Roytberg MA, Joseph Chan JWD, Rahman MS (2009). **A word counting graph**. *London Algorithmics 2008: Theory and Practice (Texts in Algorithmics)*.

[38] Karlin S, Burge C, Campbell A (1992). **Statistical analyses of counts and distributions of restriction sites in DNA sequences**. Nucleic Acids Res.

[39] Nicodème P, Salvy B, Flajolet P (2002). **Motif Statistics**. Theor Comput Sci.

[40] Durbin R, Eddy S, Krogh A, Mitchison G (1998). *Biological sequence analysis: probabilistic models of proteins and nucleic acids*.

[41] Rabin M (1963). **Probabilistic automata**. Inform Control.

[42] Salomaa A (1969). *Theory of automata*.

[43] Kucherov G, Noé L, Roytberg M (2009). **A unifying framework for seed sensitivity and its application to subset seeds**. J Bioinformatics Comput Biol.

[44] Rabiner LR (1989). **A tutorial on hidden Markov models and selected applications in speech recognition**. Proc IEEE.

[45] Aho A, Corasick M (1975). **Efficient string matching**. CACM.

[46] Regnier M, Furletova E, Roytberg MA: **An average number of suffix-prefixes**. In *Proceedings of the International Moscow Conference on computational molecular biology*. Moscow, Russia; 2009:313–314.

[47] Kulakovskiy I, Medvedeva YA, Shaefer U, Kasianov AS, Vorontsov IE, Bajic VB, Makeev VJ (2013). **HOCOMOCO: A comprehensive collection of human transcription factor binding sites models**. Nucleic Acids Res.

[48] Stormo GD (2000). **DNA binding sites: representation and discovery**. Bioinformatics.

[49] Kulakovskiy IV, Makeev VJ (2013). **DNA sequence motif: a jack of all trades for ChIP-Seq data**. Adv Protein Chem Struct Biol.

[50] Bernstein BE, Birney E, Dunham I, Green E, Gunter C, Snyder C, ENCODE Project Consortium (2012). **An integrated encyclopedia of DNA elements in the human genome**. Nature.

