# Supplementary material for: Analysis of pattern overlaps and exact computation of P-values of pattern occurrences numbers: case of Hidden Markov Models
Source: Algorithms Mol Biol. 2014 Dec 16;9:25. doi: 10.1186/s13015-014-0025-1 (PMC4307674; doi:10.1186/s13015-014-0025-1)
Supplement: Additional file 1 — Proofs of propositions. Description of data: The file contains the proofs of the propositions 2 and 3. [file 13015_2014_25_MOESM1_ESM.pdf]

Proofs of propositions

Mireille Régnier *[1,6,7], Evgenia Furletova*[2,3] , Victor Yakovlev[2,5] , Mikhail Roytberg[2,4,5]

[1] INRIA
[2] Institute of Mathematical Problems of Biology, 142290, Institutskaya, 4, Pushchino, Russia
[3] Pushchino State University, 142290, Prospect Nauki, 5, Pushchino, Russia
[4] Laboratoire J.-V. Poncelet (UMI 2615), 119002, Bolshoy Vlasyevskiy Pereulok, 11, Moscow, Russia
[5] National Research University "Higher School of Economics", 101978, Myasnitskaya str., 20, Moscow, Russia
[6] CNRS
[7] LIX-Ecole Polytechnique, 1 rue d'Estienne d'Orves, 91 120 Palaiseau, France

Email: Mireille Régnier *- mireille.regnier@inria.fr; Evgenia Furletova *- furletova@lpm.org.ru; Victor Yakovlev - v.yacovlev@gmail.com; Mikhail Roytberg - mroytberg@lpm.org.ru;

*Corresponding author

Proposition 2 Let $w \in OV(\mathcal{H})$, $w \neq \epsilon$, $s = 1, \ldots, S$. Then

$$D(n, s, w) = \cup_{x \in OverlapPrefix(w)} R(n - |Back(x, w)|, s, x) \cdot Back(x, w) \quad . \tag{1}$$

Proof: Let Y be the set described with the right side of equation (1).

1. First, we prove that $D(n, s, w) \subseteq Y$. Any text t in $D(n, s, w)$ admits the word w as a suffix and contains at least s occurrences of $\mathcal{H}$ words. Moreover, the s-th occurrence overlaps w. This overlap word, noted x, is in $OV(\mathcal{H})$: it is a suffix of a $\mathcal{H}$ word and a prefix of an $OV(\mathcal{H})$ word, hence a prefix of at least one $\mathcal{H}$ word.

   Let t' be the prefix of t ending with s-th occurrence of $\mathcal{H}$, i.e. $t = t'.Back(x, w)$. The text t' meets the following conditions:

   - it has length $n - |Back(x, w)|$;
   - it has exactly s occurrences of $\mathcal{H}$;
   - it ends with a word $\mathrm{H} \in \mathcal{H}(x)$.

   Thus $t' \in R(n - |Back(x, w)|, s, x)$ and $t \in Y$.

2. Second, we prove that $Y \subseteq D(n,s,w)$. Let $t \in Y$, i. e.

$$t \in R(n - |Back(x,w)|, s, x) \cdot Back(x,w),$$

where $x \in OverlapPrefix(w)$. By definition of R-sets:

- t is of length n;
- t contains at least s occurrences of $\mathcal{H}$; s-th occurrence is overlapping with w;
- t ends with w.

Thus $t \in D(n,s,w)$.

Q. e. d.

Proposition 3 Let $w \in OV(\mathcal{H}) \setminus \epsilon$, $n \geq m, s \geq 1$. Then

$$D(k(n,w),s,w) = D(k(n,lpred(w)),s,lpred(w)) \cdot Back(w) \bigcup R(k(n,w),s,w) \ . \tag{2}$$

Proof: Consider Proposition 2. Let

$$Y = \cup_{x \in OverlapPrefix(w) \setminus \{w\}} R(k(n,w) - |Back(x,w)|, s, x) \cdot Back(x,w).$$

Thus $D(k(n,w),s,w) = Y \cup R(k(n,w),s,w)$. Note, that:

$$OverlapPrefix(w) \setminus \{w\} = OverlapPrefix(lpred(w));$$

$$Back(x,w) = Back(x,lpred(w)) \cdot Back(w);$$

$$k(n,w) - |Back(x,w)| = k(n,lpred(w)) - |Back(x,lpred(w))|.$$

Thus $Y = D(k(n,lpred(w)),s,lpred(w)) \cdot Back(w)$.

Q. e. d.
