# Supplementary material for: Analysis of pattern overlaps and exact computation of P-values of pattern occurrences numbers: case of Hidden Markov Models
Source: Algorithms Mol Biol. 2014 Dec 16;9:25. doi: 10.1186/s13015-014-0025-1 (PMC4307674; doi:10.1186/s13015-014-0025-1)
Supplement: Additional file 2 — Algorithms of pre-processing stage of SUFPREF . Description of data: The file contains description of the algorithms used on the pre-processing stage of the algorithm SufPref. [file 13015_2014_25_MOESM2_ESM.pdf]

Algorithms of pre-processing stage of SufPref

Mireille Régnier *[1,6,7], Evgenia Furletova*[2,3] , Victor Yakovlev[2,5] , Mikhail Roytberg[2,4,5]

[1] INRIA
[2] Institute of Mathematical Problems of Biology, 142290, Institutskaya, 4, Pushchino, Russia
[3] Pushchino State University, 142290, Prospect Nauki, 5, Pushchino, Russia
[4] Laboratoire J.-V. Poncelet (UMI 2615), 119002, Bolshoy Vlasyevskiy Pereulok, 11, Moscow, Russia
[5] National Research University "Higher School of Economics", 101978, Myasnitskaya str., 20, Moscow, Russia
[6] CNRS
[7] LIX-Ecole Polytechnique, 1 rue d'Estienne d'Orves, 91 120 Palaiseau, France

Email: Mireille Régnier *- mireille.regnier@inria.fr; Evgenia Furletova *- furletova@lpm.org.ru; Victor Yakovlev - v.yacovlev@gmail.com; Mikhail Roytberg - mroytberg@lpm.org.ru;

*Corresponding author

1 Construction of the overlap graph

1.1 Aho-Corasick trie

The algorithm of *OvGraph* construction relies on the traversals of Aho-Corasick trie $T_{\mathcal{H}}$ of the pattern $\mathcal{H}$ [1]. The internal nodes of a trie $T_{\mathcal{H}}$ correspond to proper prefixes of words from the pattern $\mathcal{H}$ and the leaves correspond to the words from $\mathcal{H}$. We will identify each node (internal node or leaf) with the word corresponding to the path from the root to the node. Let t and x be two nodes of the trie. There exists a link from t to x if and only if:

1. t is the maximal proper prefix of x (prefix link, notation: $t = mpref(x)$) or
2. x is the maximal suffix of t such that x is a prefix of a word from $\mathcal{H}$ (suffix link, notation: $x = msuf(t)$).

The algorithm constructing the trie for a given set $\mathcal{H}$, and all necessary definitions are given in [1].

1.2 General description of the algorithm

To construct the overlap graph we have to construct the set of its nodes, $OV(\mathcal{H})$, and left, right and deep edges. To do this, we traverse the Aho-Corasick trie $T_{\mathcal{H}}$. First, a bottom-up traversal along the suffix links points out all vertices of $T_{\mathcal{H}}$ corresponding to words from $OV(\mathcal{H})$ and point out right edges. Then, a depth-first traversal of $T_{\mathcal{H}}$ along the prefix links creates the nodes of *OvGraph*, points out deep vertices

and creates the left and right edges. During the traversal, processed nodes of the Aho-Corasick trie are deleted; at the end of the step the trie is deleted. Finally, we create deep edges.

The first stage has time complexity $O(N_{AC}) \leq O(m \times |\mathcal{H}|)$, where N_{AC} is the number of nodes in Aho-Corasick trie. The second stage has the time complexity $O(|Q|^2 \times m \times |\mathcal{H}|)$. At this stage, we use additional memory of size $O(|Q|^2 \times (|\mathcal{H}| + |OV(\mathcal{H})|))$.

1.3 Recognition of the words $t \in OV(\mathcal{H})$ and the right edges

The sets $OV(\mathcal{H})$ and $DROV(\mathcal{H}) \subseteq OV(\mathcal{H})$ are two subsets of the set of internal nodes of the trie $T_{\mathcal{H}}$. A node t of the trie $T_{\mathcal{H}}$ belongs to $OV(\mathcal{H})$ if and only if there is a sequence of suffix links leading from a leaf to this node. A node t of the trie $T_{\mathcal{H}}$ belongs to $DROV(\mathcal{H})$ if and only if there exists a suffix link leading from a leaf to t. Let x and t be two overlap words. The pair (x, t) is a right edge (in other words, $x = rpred(t)$) if and only if a suffix link leads from t to x ($x = msuf(t)$). Therefore, a bottom-up traversal of $T_{\mathcal{H}}$ according to the suffix links allows one to reveal the nodes from $OV(\mathcal{H})$. The procedure is given in Fig. A. It marks nodes of Aho-Corasick trie corresponding to vertices of *OvGraph* and stores the information on suffix edges; this information will be used during the construction of *OvGraph*.

Algorithm 1:

Input: Aho-Corasick trie $T_{\mathcal{H}}$

Mark ϵ as an element of $OV(\mathcal{H})$;
foreach H $\in \mathcal{H}$ **do**
    $t = msuf(\mathrm{H})$;
    Store information that $rpred(\mathrm{H}) = t$; `// the information is needed for next stages, see Fig. B, C`
    while *t is not marked as an element of $OV(\mathcal{H})$* **do**
        Mark t as an element of $OV(\mathcal{H})$;
        Store information that $rpred(t) = msuf(t)$;
        $t = msuf(t)$;
    end
end

Figure A. Determination of the sets $OV(\mathcal{H})$ and $DROV(\mathcal{H})$, and right edges

1.4 Creating nodes of *OvGraph*. Determination of the set $DLOV(\mathcal{H})$ and of the left and right edges

At the previous stage, see Fig. A and section 1.3, we have already marked the nodes of Aho-Corasick trie corresponding to words $t \in OV(\mathcal{H})$ and stored information allowing to create right edges. In this section, we will describe the recursive algorithm *CrOvNodes* performing a depth-first traversal of the trie along prefix links and constructing the set $OV(\mathcal{H})$ of vertices of *OvGraph* and its left and right edges. The

construction of left edges is based on the following observation. Let x and t be two overlap words. The pair (x,t) is a left edge (in other words, $x = lpred(t)$) if and only if there exists a sequence of prefix links leading from x to t, and there are no other overlap word within the sequence. A node t of the trie $T_{\mathcal{H}}$ belongs to $DLOV(\mathcal{H})$ if and only if there exists a leaf H such that $t = lpred(\mathrm{H})$.
The algorithm $CrOvNodes$ is presented in Fig. B. Visiting a vertex t of $T_{\mathcal{H}}$ such that $t \in OV(\mathcal{H})$, the algorithm creates the corresponding node of $OvGraph$, the outgoing right edge and the incoming left edge that are incident to the node. Then the memory corresponding to the vertex t of the Aho-Corasick trie is released. At the end of the algorithm $CrOvNodes$ the Aho-Corasick trie is deleted.
Along with constructing vertices and edges of $OvGraph$ the algorithm calculates and stores some probabilities. Namely, for each node $t \in OV(\mathcal{H})$, states $\tilde{q}, q \in Q$, one computes probabilities $Prob(\tilde{q}, Back(t), q)$, and for all t in $\mathcal{H}$, one computes $Prob(\tilde{q}, Back(t), q)$ and $Prob(\tilde{q}, t, q)$. The calculation is performed following the formula (20) in the text of the paper and is analogous to the forward algorithm for HMM, see [2]. The computed probabilities are stored in the temporary data structures, the structures are not shown in Fig. B. At the end of the procedure one deletes the temporary structures used in the Algorithm 1 to store right predecessors of inner nodes of the trie (see Figure A, line 7).

Algorithm 2: *CrOvNodes*

Input: node t of $T_{\mathcal{H}}$; node *last_ov* of *OvGraph* corresponding to last overlap visited on the path leading to t during the traversal of $T_{\mathcal{H}}$; probabilities $Prob(\tilde{q}, Back(mpref(t)), q)$ and $Prob(\tilde{q}, mpref(t), q)$, $\tilde{q}, q \in Q$

// 1. Processing of node t
// 1.1. Computing of probabilities
1 **foreach** $\tilde{q}, q \in Q$ **do**
2   Compute $Prob(\tilde{q}, t, q)$;
3   Compute $Prob(\tilde{q}, Back(t), q)$; // $t = last\_ov.Back(t)$
4   **if** $t \in OV(\mathcal{H})$ **then**
5   Store $Prob(\tilde{q}, Back(t), q)$;
6   **end**
7   **if** $t \in \mathcal{H}$ **then**
8   Store $Prob(\tilde{q}, Back(t), q)$ and $Prob(\tilde{q}, t, q)$;
9   **end**
10 **end**
// 1.2. Creating of node of *OvGraph* corresponding to t
11 **if** $t \in OV(\mathcal{H})$ **then**
12   **if** $w(t)$ *was not created before* **then**
13   Create $w(t)$; // $w(t)$ is the node of *OvGraph* corresponding to t
14   **end**
15   **if** $w(rpred(t))$ *was not created before* **then**
16   Create $w(rpred(t))$; // see line 8, Fig. A
17   **end**
18   Create right edge $(w(rpred(t)), w(t))$;
19   Create left edge $(last\_ov, w(t))$;
20   Set fields of descriptor of $w(t)$; // see 3.2.2
21     $last\_ov = w(t)$;
22 **end**
23 **if** $t \in \mathcal{H}$ **then**
24   Mark *last_ov* as an element of $DLOV(\mathcal{H})$;
25   Store information that $lpred(t) = last\_ov$; // the information is needed to create deep edges, see Fig. C
26   **if** $w(rpred(t))$ *was not created before* **then**
27   Create $w(rpred(t))$;
28   **end**
29   Mark $w(rpred(t))$ as an element of $DROV(\mathcal{H})$;
30 **end**
// 2. Recursion. Depth-first traversal of $T_{\mathcal{H}}$ along prefix links
31 **foreach** x *such that* $t = mpref(x)$ **do**
32     $CrOvNodes(x, last\_ov$, probabilities $Prob(\tilde{q}, Back(t), q)$ and $Prob(\tilde{q}, t, q)$, $\tilde{q}, q \in Q)$;
33 **end**
// 3. Release memory corresponding to t
34 Delete t from $T_{\mathcal{H}}$;

Figure B. Procedure *CrOvNodes*. Creating of nodes of *OvGraph*, left and right edges. Determination of the set $DLOV(\mathcal{H})$. At the first call of the algorithm, the input node t is the root of the trie. The node of *OvGraph* corresponding to a node t of Aho-Corasick trie is denoted as $w(t)$, see lines 12-21, 26-29.

1.5 Construction of overlap classes and deep edges

The algorithm is shown in Fig. C. Using a depth-first traversal of *OvGraph* along left edges, it looks over all left deep nodes. During the processing of left deep nodes (lines 2-15), it assigns to each word $\mathrm{H} \in \mathcal{H}$ the identifier of its overlap class $\mathrm{H}.CLASS$ (line 6 or line 9) and creates the deep edges (see line 12). For each right deep node r, $lpred(\mathrm{H})$ of the last visited word H in $\mathcal{H}$ such that $rpred(\mathrm{H}) = r$ is memorized in a field $r.LOG$; the identifier of the overlap class of H is memorized in $r.CLASS$. Initially the fields are set to NIL. The Aho-Corasick trie being already deleted, we use the temporary structures created by the algorithms given in Fig. A and B. Namely, the structures store, for each $\mathrm{H} \in \mathcal{H}$, links to $lpred(\mathrm{H})$ and $rpred(\mathrm{H})$. The run-time is $O(|\mathcal{H}| + |OV(\mathcal{H})|)$.

Algorithm 3:

1 $NumClasses := 0$;
2 **foreach** *left deep nodes* $w \in OV(\mathcal{H})$ *in order of depth-first traversal of OvGraph* **do**
3   **foreach** $\mathrm{H} \in \mathcal{H}$ *such that* $w = lpred(\mathrm{H})$ **do**
4         $r := rpred(\mathrm{H})$;
5   **if** $r.LOG = w$ **then**
6             $\mathrm{H}.CLASS := r.CLASS$; `// add H to the class` $\mathrm{H}^*(w, r)$;
7   **else**
8             $NumClasses := NumClasses + 1$;
9             $\mathrm{H}.CLASS := NumClasses$; `// create class` $\mathrm{H}^*(w, r)$;
10             $r.CLASS := \mathrm{H}.CLASS$; `// rewrite parameters for` r;
11             $r.LOG := w$;
12   Create deep edge (w, r);
13   **end**
14   **end**
15 **end**

Figure C. Creating overlap classes and deep edges

2 Sets of states associated to the nodes of *OvGraph* and transition probabilities

At the preprocessing stage of the algorithm SufPref we construct the sets of states $AllState(w)$ and $PriorState(w, q)$, for each $w \in OV(\mathcal{H})$ and $q \in AllState(w)$, and compute the following probabilities:

- left transition probabilities $Prob(\tilde{q}, Back(w), q)$, where $q \in AllState(w)$ and $\tilde{q} \in PriorState(w, q)$;
- deep transition probabilities $Prob(\tilde{q}, Back(\mathrm{H}^*(w, r)), q)$, where $w \in DLOV(\mathcal{H})$ and $r \in DROV(\mathcal{H})$, $q \in AllState(r)$ and $\tilde{q} \in PriorState(\mathrm{H}^*(w, r), q)$;
- word probabilities $Prob(\tilde{q}, \widetilde{\mathcal{H}}(r), q)$, where $r \in DROV(\mathcal{H})$, $q \in AllState(r)$ and $\tilde{q} \in Q$.

The procedure is given in the Figure D.

Remark:

1. To be more precise, it is enough to compute deep transition probabilities $Prob(\tilde{q}, \mathrm{H}^*(w,r), q)$ only for such pairs $(\tilde{q}, q)$ that exists $\mathrm{H} \in \mathrm{H}^*(w,r)$ where $\tilde{q} \in PriorState(\mathrm{H})$ and $q \in AllState(\mathrm{H})$. However, r is a suffix of all words $\mathrm{H} \in \mathrm{H}^*(w,r)$, therefore

$$\bigcup_{\mathrm{H}\in \mathrm{H}^*(w,r)} AllState(\mathrm{H}) \subseteq AllState(r).$$

Computing all deep transition probabilities, we possibly compute zero probabilities but simplify the program. Analogously, we compute word probabilities $Prob(\tilde{q}, \widetilde{\mathcal{H}}(r), q)$ for all $q \in AllState(r)$.

2. After the running of procedures given in Fig. A-C, one stores, for each $\mathrm{H} \in \mathcal{H}$: links to $lpred(\mathrm{H})$ and $rpred(\mathrm{H})$, the probabilities described above and the number of the corresponding overlap class. This information allows one to create for all $r \in DROV(\mathcal{H})$ the list of identificators of words $\mathrm{H} \in \mathcal{H}$ such that $r = rpred(\mathrm{H})$ (it is used in line 18, see Fig. D); and for each overlap class the list of identificators of words that are in the class (it is used in line 27, see Fig. D). After the end of the procedure the memory needed to store the information can be released.

Algorithm 4:

Input: *OvGraph*; probabilities $Prob(\tilde{q}, Back(w), q)$, $Prob(\tilde{q}, Back(\mathrm{H}), q)$ and $Prob(\tilde{q}, \mathrm{H}, q)$ where $\tilde{q}, q \in Q$, $w \in OV(\mathcal{H})$ and $\mathrm{H} \in \mathcal{H}$

foreach *node* w *of OvGraph by depth-first traversal of left edges* **do**

// A.Constructing of sets of states $AllState(w)$, $PriorState(w,q)$, list of left transition probabilities $Prob(\tilde{q}, Back(w), q)$, where $q \in AllState(w)$ and $\tilde{q} \in PriorState(w,q)$

// Initialization

if $w = \epsilon$ **then**

$AllState(\epsilon) := Q$;

foreach $q \in Q$ **do**

$PriorState(\epsilon, q) := \emptyset$;

end

else

foreach $q \in Q, \tilde{q} \in AllState(lpred(w))$ **do**

if $Prob(\tilde{q}, Back(w), q) > 0$ **then**

Add q to $AllState(w)$;

Add $\tilde{q}$ to $PriorState(w,q)$;

Add $Prob(\tilde{q}, Back(w), q)$ to the list of left transition probabilities assigned to w;

end

end

end

// B. Constructing of list of word probabilities $Prob(\tilde{q}, \widetilde{\mathcal{H}}(w), q)$, where $w \in DROV(\mathcal{H})$, $q \in AllState(w)$ and $\tilde{q} \in Q$

if $w \in DROV(\mathcal{H})$ **then**

foreach $q \in AllState(w), \tilde{q} \in Q$ **do**

Compute $Prob(\tilde{q}, \widetilde{\mathcal{H}}(w), q)$ by summation of $Prob(\tilde{q}, \mathrm{H}, q)$ over all H such that $w = rpred(\mathrm{H})$;

if $Prob(\tilde{q}, \widetilde{\mathcal{H}}(w), q) > 0$ **then**

Add $Prob(\tilde{q}, \widetilde{\mathcal{H}}(w), q)$ to the list of word probabilities assigned to w;

end

end

end

end

// C.Constructing of list of deep transition probabilities $Prob(\tilde{q}, Back(\mathrm{H}^*(w,r)), q)$, where $w \in DLOV(\mathcal{H})$ and $r \in DROV(\mathcal{H})$, $q \in AllState(r)$ and $\tilde{q} \in PriorState(\mathrm{H}^*(w,r), q)$

foreach $\mathrm{H}^*(w,r) \in \mathcal{P}(\mathcal{H})$ **do**

foreach $q \in AllState(r), \tilde{q} \in AllState(w)$ **do**

Compute $Prob(\tilde{q}, Back(\mathrm{H}^*(w,r)), q)$ by summation of $Prob(\tilde{q}, Back(\mathrm{H}), q)$ over all $\mathrm{H} \in \mathrm{H}^*(w,r)$;

if $Prob(\tilde{q}, Back(\mathrm{H}^*(w,r)), q) > 0$ **then**

Add $\tilde{q}$ to $PriorState(\mathrm{H}^*(w,r), q)$;

Add $Prob(\tilde{q}, Back(\mathrm{H}^*(w,r)), q)$ to the list of deep transition probabilities assigned to r;

end

end

end

Figure D. Creating of sets of states $AllState(w)$ and $PriorState(w,q)$, $w \in OV(\mathcal{H}), q \in AllState(w)$, and creating of lists with left transition probabilities, deep transition probabilities and word probabilities

The run-time of the procedure is $O(|Q|^2 \times (|OV(\mathcal{H})| + |\mathcal{H}|))$.

References

1. Aho A, Corasick M: **Efficient string matching**. *CACM* 1975, **18**(6):333–340.

2. Durbin R, Eddy S, Krogh A, Mitchison G: *Biological sequence analysis: probabilistic models of proteins and nucleic acids*. Cambridge: Cambridge University 1998.
